# A phase II trial protocol of Tocilizumab in anti-TNF refractory patients with JIA-associated uveitis (the APTITUDE trial)

**DOI:** 10.1186/s41927-018-0010-2

**Published:** 2018-02-27

**Authors:** Athimalaipet V. Ramanan, Andrew D. Dick, Ashley P. Jones, Catherine Guly, Ben Hardwick, Helen Hickey, Richard Lee, Andrew McKay, Michael W. Beresford, The APTITUDE Trial Management Group

**Affiliations:** 1 University Hospitals Bristol NHS Foundation Trust & Bristol Medical School, University of Bristol, Bristol, UK; 2 Bristol Eye Hospital, Bristol, UK; 3 School of Clinical Sciences, University of Bristol, UCL Institute of Ophthalmology and National Institute for Health Research Biomedical Research Centre at Moorfields Eye Hospital and University College London Institute of Ophthalmology, Bristol, London UK; 4 Clinical Trials Research Centre, Department of Biostatistics, University of Liverpool, Liverpool, UK; 5 Department of Women’s and Children’s, Institute of Translational Medicine, University of Liverpool, Liverpool, UK; 6 Department of Paediatric Rheumatology, Alder Hey Children’s NHS Foundation Trust, Liverpool, UK

**Keywords:** Juvenile Idiopathic Arthritis, Uveitis, Ophthalmology, Rheumatology, Paediatric, Tocilizumab, Methotrexate, Safety

## Abstract

**Background:**

Juvenile idiopathic arthritis (JIA) is the most common rheumatic disease in children. Children with JIA are at risk of intraocular inflammation (uveitis). In the initial stages of mild-moderate inflammation uveitis is asymptomatic. Most children with mild-moderate uveitis are managed on topical steroid drops with or without systemic methotrexate (MTX). When children with moderate-severe uveitis are refractory to MTX, monoclonal anti-tumour necrosis factor agents have been trialled, interim analysis data showed positive results. However, several children with severe recalcitrant disease or non-responsive to anti-tumour necrosis factor agents remain and are at greater risk of significant ocular complications and visual loss. Further evidence of alternative therapies is needed with evidence of a potential role of anti-interleukin-6 agents in the management of severe refractory uveitis.

**Methods:**

The trial will be conducted following a two-stage Simon design. The trial will register at least 22 patients aged 2 to 18 years with active JIA-associated uveitis, who have taken MTX for at least 12 weeks and have failed an anti-TNF agent. It will take place in 7 centres across the UK. All participants will be treated for 6 months, with follow up of 9 months from registration. Participants will receive a stable dose of MTX and those weighing ≥30 kg will be dosed with 162 mg of Tocilizumab every 2 weeks and participants weighing < 30 kg dosed with 162 mg of Tocilizumab every 3 weeks. Primary outcome is treatment response at 12 weeks. Adverse events will be collected up to 30 calendar days following treatment cessation.

**Discussion:**

This is a novel adaptive design study of subcutaneous IL-6 inhibition in anti-TNF refractory JIA associated uveitis which will be able to determine if further research should be conducted. This is the first trial to look at ophthalmology outcomes in the efficacy of Tocilizumab in uveitis.

This is the first paediatric clinical trial to assess the clinical effectiveness and safety of tocilizumab with MTX in JIA associated uveitis.

**Trials registration:**

The Trial is registered on the ISRCTN registry (ISRCTN95363507) on the 10/06/2015 and EU Clinical Trials Register on the 03/07/2015 (EudraCT Number: 2015–001323-23).

**Electronic supplementary material:**

The online version of this article (10.1186/s41927-018-0010-2) contains supplementary material, which is available to authorized users.

## Background

Juvenile idiopathic arthritis (JIA) is the name for a type of arthritis that primarily affects young people and whose cause is unknown. Although ‘arthritis’ refers to inflammation of the joints, in JIA the inflammation may also affect the eyes and other internal organs. Approximately 1 in 1000 children in the UK develops JIA per annum. Although both genders are affected, JIA is most common in girls. JIA is the most common rheumatic disease in children. Children with JIA also are at risk of inflammation of the uvea in the eye (uveitis). 80% of all paediatric uveitis is secondary to JIA [1, 2]. The major risk factors for development of uveitis in JIA are oligoarticular pattern of arthritis, early onset of arthritis, and antinuclear antibody positivity [3]. In the initial stages of mild to moderate inflammation the uveitis is entirely asymptomatic.

Most children with mild to moderate uveitis are managed on topical steroid drops and systemic methotrexate (MTX) as an immunosuppressive agent [4, 5]. As a significant proportion of children with moderate-severe uveitis are refractory to MTX [6–8], biologics in the form of monoclonal anti-TNF agents have been tried. The anti-TNF agents are effective only in 30–60% of the patients based on several retrospective case series [9]. When children with moderate-severe uveitis are refractory to MTX, monoclonal anti-TNF agents have been trialled [10] the results show that adalimumab in combination with methotrexate was effective at treating JIA-associated uveitis [11]. Unfortunately, anti-tumour necrosis factor (anti-TNF) therapy is not the panacea either for JIA alone, or JIA-associated uveitis. We know that the uveitis in some patients may still flare despite anti-TNF therapy. These children have severe recalcitrant disease that is therefore at greater risk of causing significant ocular complications and blindness.

Interleukin 6 (IL-6), glycoprotein 130, and IL-6 receptor levels are all elevated in uveitis in many studies of sampling of ocular fluids/tissues [12]. The cause of chronic disease remains unknown but auto-inflammation driving a dysregulated innate immunity will encompass an IL-6 mediated inflammatory response, particularly from mononuclear cell populations and is a consistent feature of animal models and human data to date [13]. As such, IL-6 is a suitable inflammatory pathway to therapeutically target. Not surprisingly, there are anecdotal reports of success of using anti-IL-6 therapy in the form of tocilizumab, in refractory uveitis [14–16].

### Rationale

In view of the failure of patients with refractory JIA-associated uveitis to either respond, or subsequently flare on anti-TNF therapy, and the strong evidence base for the rationale for targeting IL-6 in the disease pathogenesis, a phase II trial of the potential efficacy, safety and tolerability of anti-IL-6 therapy is being undertaken to decide whether further research is justified. Previous studies investigating the effect of Tocilizumab in paediatric arthritis have excluded patients with uveitis. However, data available for Tocilizumab used in treating uveitis in adults indicates its potential role for refractory disease [17]. A systematic search of existing data has shown limited case reports [18].

Tocilizumab (trade name RoActemra) is a biological therapy blocking the action of IL-6. In arthritis, high concentrations of IL-6 are associated with tiredness, anaemia, and inflammation and damage to bones, cartilage and tissue. Tocilizumab reduces these effects. Previous clinical trials of Tocilizumab in children with systemic-onset JIA have shown that children respond dramatically to treatment in a short time span [19]. Tocilizumab obtained National Institute for Health and Care Excellence approval for this indication and became the first drug to be licenced for the use of JIA in fifty years. The trial in polyarticular forms of JIA shows a good effect [20]. However these clinical trials for Tocilizumab state that a diagnosis of uveitis is part of the exclusion criteria and therefore no indication of efficacy in uveitis can be determined.

Tocilizumab is now the first choice biologic in children with systemic onset JIA and an important agent in the management of refractory polyarticular JIA. An ongoing Phase 1b, open-label, multicenter study to investigate the pharmacokinetics, and safety of Tocilizumab following subcutaneous administration to patient with polyarticular-course juvenile idiopathic arthritis is currently underway across 13 countries worldwide [21].

### Potential risks and benefits

However, due care must be taken in determining the potential benefits of Tocilizumab therapy against the potential associated risks, and as such, the risk / benefit assessment of this intervention and safety is a key secondary outcome measure of the trial.

The most commonly reported Adverse Drug Reactions (ADRs) (occurring in ≥5% of patients treated with Tocilizumab monotherapy or in combination with disease-modifying anti-rheumatic drugs) were upper respiratory tract infections, nasopharyngitis, headache, hypertension and increased alanine aminotransferase test (ALT). The most serious ADRs were serious infections, complications of diverticulitis, and hypersensitivity reactions [19, 20].

Less likely in a paediatric population, rheumatoid arthritis patients have an increased risk of cardiovascular disorders such as hypertension or hyperlipemia. There are no known evidence that Tocilizumab treatment effects demyelinating disorders in a paediatric population. There are risks of local site reactions, including erythema, pain, induration and subcutaneous emphysema among others.

Of note, Tocilizumab has been shown to reduce the rate of progression of joint damage as measured by X-ray and to improve physical function when given in combination with methotrexate [20]. Tocilizumab treatment has been shown in a randomized clinical trial of polyarticular-course JIA, as well as systemic-onset JIA, as demonstrating significant improvement, maintained over time, of signs and symptoms and has a safety profile consistent with that for adults with RA [20].

In view of these data, we aimed to develop the protocol and deliver the first paediatric clinical trial that will assess the clinical effectiveness and safety of tocilizumab in combination with MTX for the treatment of anti-tumour necrosis factor refractory JIA-associated uveitis. The protocol for this trial is presented here.

## Methods

### Trial design

**Fig. 1:**
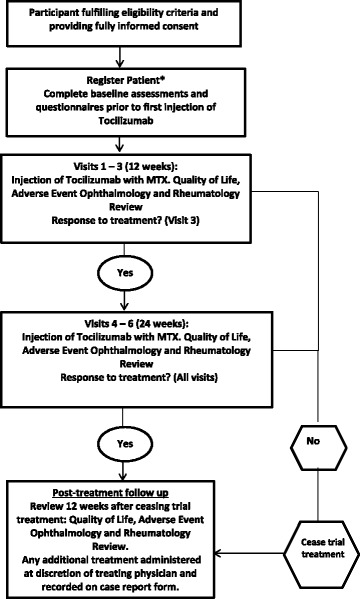
Schematic of Study Design. *Registration should take place no later than 2 weeks after the beginning of screening

APTITUDE is a single arm Phase II trial that will be conducted following a two-stage Simon design [20]. Figure 1 outlines the schematic of trial design and patient journey. All participants will be treated for 6 months, with follow-up after 3 months of treatment cessation (continuing on MTX throughout). Recruitment started on 3rd December 2015.

All participants will complete trial assessments as shown in Table 1. Further details are available on the trial’s website [22].

**Table 1:** Study visits and assessments

Assessment (Procedure/ Activity	Screening	Baseline	Visit 1	Visit 2	Visit 3	Visit 4	Visit 5	Visit 6	Visit 7
Weeks		0 (−7/+ 7 days)	4 (− 7/+ 7 days)	8 (− 7/+ 7 days	12 (− 7/+ 7 days)	16 − 7/+ 7 days)	20 (− 7/+ 7 days)	24 (− 7/+ 7 days)	36 (− 7/+ 7 days)
	Screening	Baseline/ Registration	Study Visit	Study Visit	Assessment of endpoints	Study Visit	Study Visit	End of treatment	End of trial
Written and informed consent	X								
Confirm consent	X	X	X	X	X	X	X	X	X
Assessment of eligibility criteria	X	X							
Review of Medical/ Ophthalmic/ Surgical History	X								
Review of concomitant medications	X	X	X	X	X	X	X	X	X
Pregnancy test	X		X	X	X	X	X	X	X
Purified protein derivative Tuberculin Skin Test/ Test latent Tuberculosis as locally performed	X								
Urinalysis	X	X	X	X	X	X	X	X	X
Study intervention (Injection)		X	X	X	X	X	X	X	
Compliance with study intervention		X	X	X	X	X	X	X	
Physical Examination		X	X	X	X	X	X	X	X
Vital signs (heart and respiratory rate and blood pressure)	X		X	X	X	X	X	X	X
Height/ Weight	X	X	X	X	X	X	X	X	X
Dispense treatment diary		X	X	X	X	X	X	X	
Child Health Questionnaire		X	X	X	X	X	X	X	X
Childhood Health Assessment Questionnaire		X	X	X	X	X	X	X	X
Haematological analysis	X	X*	X	X	X	X	X	X	X
Biochemical analysis	X	X*	X	X	X	X	X	X	X
Anti-nuclear antibodies, double stranded deoxyribonucleic acid and extractable nuclear antigens		X						X	
Samples for Biobank		X			X				X
Vision Assessments^#^	X	X	X	X	X	X	X	X	X
Optical coherence tomography	X		X	X	X	X	X	X	X
Anterior Chamber cells and flare assessment^#^	X	X	X	X	X	X	X	X	X
Assessment of vitritis and vitreous haze	X		X	X	X	X	X	X	X
Cataract scoring	X		X	X	X	X	X	X	X
Goldman tonometry or tonopen	X		X	X	X	X	X	X	X
Standard American College of Rheumatology Pediatric Score Set Outcome Variables		X	X	X	X	X	X	X	X
Tanner Score		X			X			X	X
Assessments of Adverse and Serious Adverse Events		X	X	X	X	X	X		

* Biochemical and Haematological taken at screening can be used at baseline only if taken with 2 weeks of baseline visit

^#^ Tests do not need to be repeated if screening and baseline visit occurs on the same day

### Study population

Children aged between 2 and less than 18 are eligible for the trial and at screening must have active uveitis defined as “2” readings of cellular infiltrate in anterior chamber of Standardisation of the Uveitis Nomenclature (SUN) criteria [23] grade ≥ 1+ or more during the preceding 6 weeks. Participants must have failed an anti-TNF agent and have been on at least one anti-TNF agent regardless of dose for at least 12 weeks at any time previously and must have been on MTX for at least 12 weeks (stable dose for 4 weeks prior to screening) and failed. Participants of reproductive potential (males and females), must use a reliable means of contraception throughout their trial participation and post pubertal females must have a negative serum pregnancy test within 14 days prior to registration. Exclusion criteria are summarised in Table 2.

**Table 2:** Exclusion Criteria

Exclusion Criteria	
Uveitis without a diagnosis of JIA fulfilling International League if Associations for Rheumatology diagnostic criteria for JIA (all subgroups that have uveitis).	Currently on Tocilizumab or has previously received Tocilizumab.
Previous registration into the APTITUDE trial.	Participation in another clinical trial of investigational medicinal product within the last 4 weeks or 5 serum half-lives (whichever is longer) prior to registration.
More than 6 topical steroid eye drops per day per eye at time of registration (dose must be stable for 1 week prior to registration).	For participants on Prednisone or Prednisone equivalent, change of dose within 4 weeks prior to registration.
Participants on prednisone or prednisone equivalent with a dose > 0.2 mg/kg per day.	No intraocular injection of disease modification agents including steroids and anti-Vascular endothelial growth factor within 4 weeks prior to registration.
No intraocular surgery for previous 12 weeks prior to registration or expected/panned for duration of study.	Lack of recovery from recent surgery or surgery within 6 weeks at the time of registration.
Intra-ocular pressure ≥ 25 mmHg at time of registration.	Participants requiring systemic therapy with oral anti-glaucoma medication.
No disease modifying immunosuppressive drugs, other than MTX in the 4 weeks prior to registration	History of active tuberculosis of less than 24 weeks treatment.
Latent tuberculosis not successfully treated for at least 4 weeks prior to registration (a test for latent tuberculosis infection must be performed within 12 weeks prior to registration).	Auto-immune, rheumatic disease or overlap syndrome other than JIA.
Females who are pregnant, lactating, or intending to become pregnant during trial.	Known human immunodeficiency virus infection or other condition characterized by a compromised immune system.
Any history of alcohol or drug abuse within 24 weeks prior to registration.	Any active acute, sub-acute, chronic, or recurrent bacterial, viral, systemic fungal, infection or any major episode of infection requiring hospitalisation or treatment with IV antibiotics within 4 weeks of registration or treatment with oral antibiotics within 2 weeks of registration.
History of reactivation or new onset of a systemic infection such as herpes zoster or Epstein−Barr virus within 8 weeks prior to registration.	Hepatitis B surface antigen or hepatitis C antibody positivity or chronic viral or autoimmune hepatitis.
History of concurrent serious gastrointestinal disorders.	Evidence of current serious uncontrolled concomitant cardiovascular (including hyperlipidaemia), nervous system, pulmonary (including obstructive pulmonary disease), renal and hepatic disease.
History of or current cancer or lymphoma.	Persistently poorly controlled severe hypertension (>95th percentile for height / age).
Uncontrolled diabetes mellitus.	History of severe allergic or anaphylactic reactions to human, humanized or murine monoclonal antibodies.
No live attenuated vaccines (including seasonal nasal flu vaccine, varicella vaccine for shingles or chickenpox, measles, mumps and rubella (MMR) or MMR varicella, oral polio vaccine and vaccines for yellow fever, measles, mumps or rubella) 4 weeks prior to registration, throughout the duration of the trial and for 8 weeks following the last dose of study drug.	Previous treatment with any cell-depleting therapies, including investigational agents or approved therapies.
Treatment with intravenous gamma globulin or plasmapheresis within 24 weeks of registration	Any previous treatment with alkylating agents such as chlorambucil, or with total lymphoid irradiation
Any significant medical or surgical condition that would risk the participant’s safety or their ability to complete the trial	Any joint injections within 4 weeks prior to registration
Any psychological condition that in the opinion of the principal investigator would interfere with safe completion of the trial	Demonstrations of clinically significant deviations from the following laboratory parameters: Serum creatinine > 1.5 × the upper limit of normal (ULN) for age and sex Aspartate Aminotransferase Test or ALT > 1.5 × the ULN for age and sex Total bilirubin > 1.3 mg/dL (> 23 μmol/L) Platelet count < 150 × 10^3^/μL (< 150,000/mm3) (< 150 × 10^9^/L) Haemoglobin < 7.0 g/dL (< 4.3 mmol/L) White blood cell (WBC) count < 4000/mm^3^ (< 4.0 × 10^9^/L) Neutrophil count < 2000/mm^3^ (< 2.0 × 10^9^/L)

### Selection of centres

Criteria for the selection of centres will be determined by the Trial Management Group based also on track record of the centre recruitment to previous trials, including trial of anti-TNF in JIA-associated uveitis [10]. Initiation of centres will be undertaken in compliance with the Clinical Trial Research Centre (CTRC) Standard Operating Procedures. Centres fulfilling the criteria will be selected to be recruitment centres for the APTITUDE trial and will be opened to recruitment upon successful completion of all global and study-specific conditions and once all necessary documents have been returned to CTRC.

### Interventions

Patients will receive Tocilizumab dosed according to body weight, with patients weighing ≥30 kg dosed with 162 mg of Tocilizumab every 2 weeks and patients weighing < 30 kg dosed with 162 mg of Tocilizumab every 3 weeks. Trial intervention should be given up to and including week 24. Patients weighing < 30 kg will be given a maximum of 9 injections and patients weighing ≥30 kg will be given a maximum of 13 injections.

### Treatment adherence

Participants who miss two consecutive doses or three doses in total of Tocilizumab injection should cease trial treatment and will be recorded as a withdrawal from treatment.

### Concomitant therapy

A complete list of concomitant therapies that are allowed during the course of the trial and those that are not permitted can be found below.

The following medications are permitted:

- Low dose of steroids (≤0.2 mg/kg/day of prednisone or prednisolone equivalent medication orally) are permitted during the active phase of the trial.
- Topical steroid eye drops with maximum of 6 drops/ day at registration
- Failure to reduce eye drops to 2 drops/ day by or at the 12 week visit will be considered a treatment failure and the participant should cease trial treatment.
- After 12 weeks topical treatment must be kept at twice per day.
- Intraocular pressure medication apart from systemic treatment with acetazolamide
- Maxidex, Predforte or equivalent – preparation to be stipulated at screening and to remain unchanged for individual throughout treatment-phase of trial
- Intra-articular joint injections

The following medications are not permitted:

- Intra-ocular or peri-ocular corticosteroid injection
- The introduction of oral steroids, or increase in oral steroids, is not permitted at any time during the trial.
- Intravenous methylprednisolone at any time
- Other biologic therapies, including: etanercept, infliximab, golimumab; rituximab, abatacept, anakinra.
- Cyclosporine, mycophenolate mofetil, azathioprine, lefunamide, sulfasalazine, hydroxychloroquine, any other disease modifying, anti-rheumatic drug
- Systemic treatment with acetazolamide

### Registration

Registration will be undertaken by delegated individuals at trial sites. Participants will be registered using a secure (24-h) web based registration programme.

### Assessments and procedures

As described previously [10], after obtaining written consent (and assent where appropriate) from the participant, parent or legal guardian, a medical/ophthalmic history will be taken and recorded on the appropriate case report form (CRF) with particular emphasis on other disorders of relevance and allergies.

Separate sections on the CRF will be provided to record the JIA and uveitis specific medical/ophthalmic history and the participant’s other medical/surgical history. Medication (prescription, over-the-counter, and herbal supplements) use over the 4 weeks prior to the screening visit will also be recorded. Physical examination, measures of disease activity and complications, medication history, surgical history and laboratory tests (haematological and biochemical analysis and urinalysis) will be performed at the screening visit and will be repeated at each subsequent trial visit (see Table 1).

The visits are calculated from the date of the first dose of investigational medicinal product (IMP). With regards to treatment timelines, 1 month’s treatment is defined as 4 weeks; after commencing treatment the dates of each subsequent visit should be made at four weekly intervals from the date of the first dose of IMP. A window of ±7 days is allowed for these monthly visits, however for determining treatment response there should be an interval of at least 3 weeks between assessments. This means that if a patient attends a visit late (within the + 7 days window) then their next visit must not be early. The next visit must either be on the date as scheduled applying the 4 weekly intervals from first dose of IMP, or may be in the + 7 day period.

### Primary endpoint

The primary endpoint is response after 12 weeks of treatment.

Response to treatment is defined as per SUN criteria [23] as a 2 step decrease in the level of inflammation (anterior chamber cells) or decrease to zero between baseline (prior to trial treatment initiation) and 12 weeks of treatment.

### Secondary endpoints

A complete list of secondary endpoints can be found below.

1) Safety, tolerability and compliance

- Adverse events, serious adverse events and adverse events of special interest
- Laboratory parameters (haematological and biochemical analysis and urinalysis)
- Participant diaries and dosing records will determine tolerability and compliance throughout the trial treatment period

2) Use of corticosteroids over duration of study period and throughout follow up, including:

- Total oral corticosteroid dose
- Reduction in and rate of systemic corticosteroid dose from entry dose
- Topical corticosteroid use (frequency) compared to usage at registration.

3) Optic and Ocular

- Visual acuity measured by age-appropriate logarithm of the minimum angle of resolution assessment
- Number of participants with resolution of associated optic nerve or macular oedema (as assessed by slit lamp bio microscopy or optical coherence tomography).
- Number of participants who are able to reduce topical or systemic agents for ocular hypertension.
- Number of participants with disease control (defined as zero cells, with topical treatment at 12 weeks treatment visit and 24 weeks treatment visit.)
- Number of participants entering disease remission (defined as zero cells, without topical treatment at 12 and 24 weeks treatment visit)
- Duration of sustaining inactive disease (zero cells, with or without topical treatment.)
- Failure to reduce eye drops to 2 drops/day by or at the 12 weeks visit

4) Quality of Life assessment (Childhood Health Questionnaire [24], (Childhood Health Assessment Questionnaire [25]).

5) American College of Rheumatology (ACR) Pedi core set criteria [26]: at ACR30, ACR50, ACR70, ACR90 and ACR100 levels.

6) Number of participants requiring change in biologic / Disease-modifying anti-rheumatic drugs therapy due to disease flare of their arthritis or failure to respond to treatment for their arthritis.

7) Number of participants undergoing flare of arthritis [27], in remissions on and off medication of their Juvenile Idiopathic Arthritis [28] and with minimum disease activity [29].

8) Participants score of the Juvenile Arthritis Disease Activity Score (JADAS) [30]. The JADAS comprises four components: physician global assessment of disease activity parent/patient global assessment of well-being active joint count, in 27, 71 or 10 joints; and (27) erythrocyte sedimentation rate.

### Sample size

The trial will be conducted following a two-stage Simon design [31]. The null hypothesis (response = 20%) reflects a response rate of no clinical benefit while the alternative hypothesis (response = 50%) reflects a desired response. The interim and final sample sizes and the critical values for abandoning Tocilizumab at each stage have been chosen to achieve the following properties. If the true success probability is 0.2 then we will recommend further study of Tocilizumab with probability less than 0.05 (falsely pursuing a non-promising therapy). If the true success probability is 0.5 then we will recommend further study of Tocilizumab with probability greater than 0.9 (correctly pursuing a promising therapy).

After 10 participants have completed their 3 month follow up there will be an interim analysis. If there are 8 or more non-treatment responses then the trial will stop with the conclusion that the study of Tocilizumab should be abandoned. If there are fewer than 8 non-treatment responses then the study will continue until a further 12 participants have received treatment, giving a total sample size of 22. If amongst these 22 participants there are 15 or more non-treatment responses then it will be concluded that the further study of Tocilizumab should be abandoned. If further study of the drug is not abandoned at either the interim of the final analysis, then a recommendation to conduct a comparative, randomised phase III trial will be made.

Formal interim analyses of the accumulating data will be performed at regular intervals (as described above) for review by an Independent Data Monitoring and Safety Committee (IDSMC). The IDSMC will make recommendations to the Trial Steering Committee as to the continuation of the trial.

### Analysis plan

The analysis will be carried out according to the pre-defined statistical analysis plan, which can be seen in Additional file 1. If consent to treatment is withdrawn but the participant agrees to remain in the study for follow-up, the participant will be followed until completion. If the participant decides to withdraw consent completely, however, the reasons for withdrawal of consent will be recorded (if possible) and reported.

The primary outcome is ‘response to treatment’ and in the final analysis, point estimates and confidence intervals will be computed using the method described by Jovic and Whitehead [32].

### Study organisation and funding

APTITUDE is co-ordinated from the CTRC at the University of Liverpool and is sponsored by University Hospitals Bristol NHS Foundation Trust, and has been developed by the UK’s Experimental Arthritis Treatment Centre for Children [33]. It is an independent trial funded by Arthritis Research UK. The trial has a Trial Steering committee (which compromises of independent members as well as the Chief Investigators, namely AR and MWB) and an Independent Data and Safety Monitoring Committee. The IMP is provided by Roche Products Limited.

## Discussion

This trial is a novel adaptive design study of subcutaneous IL-6 inhibition in anti-TNF refractory JIA associated uveitis which will be able to determine if further research into the use of this intervention for the treatment of anti-TNF refractory JIA-associated uveitis should be conducted. This is the first trial to look at ophthalmology outcomes in regards to the efficacy of Tocilizumab in uveitis. This is the first paediatric clinical trial that will assess the clinical effectiveness and safety of tocilizumab in combination with MTX for the treatment of anti-tumour necrosis factor refractory JIA-associated uveitis. The results of this study will help decide if further investigation into the effectiveness of tocilizumab is warranted in future clinical trials.

### Trial status

This summary is based on version 4.0 of the study protocol, dated 31st May 2017.

At the time of submission this trial is open in 7 Hospital sites and has recruited 19 participants.

## Additional file

Additional file 1: Statistical analysis plan (PDF 607 kb)

## Abbreviations

ADR: Adverse Drug Reaction
ALT: Alanine Aminotransferase Test
CRF: Case Report Form
CTRC: Clinical Trials Research Centre
IDSMC: Independent data and Safety Monitoring Committee
IL-6: Interleukin-6
IMP: Investigational Medicinal Product
JIA: Juvenile idiopathic arthritis
MMR: Measles, mumps and rubella
MTX: Methotrexate
RA: Rheumatoid Arthritis
SUN: Standardisation of the Uveitis Nomenclature
TNF: Tumour necrosis factors
ULN: Upper Limit of Normal
