# Supplementary material for: A phase II trial protocol of Tocilizumab in anti-TNF refractory patients with JIA-associated uveitis (the APTITUDE trial)
Source: BMC Rheumatol. 2018 Feb 27;2:4. doi: 10.1186/s41927-018-0010-2 (PMC6390576; doi:10.1186/s41927-018-0010-2)
Supplement: Supplementary file 1 — Statistical analysis plan (PDF 607 kb) [file 41927_2018_10_MOESM1_ESM.pdf]

APTITUDE A phase II trial of Tocilizumab in anti-TNF refractory patients with JIA associated uveitis.

EudraCT number: 2015-001323-23

ISRCTN number: 95363507

AR UK Ref: 20659

Sponsor Ref: CH/2013/4247

Statistical Analysis Plan – Final Analysis

	ORIGINATED BY	QC PERFORMED BY	APPROVED BY
**Name**	Ashley Jones	TBC	Ashley Jones
**Title**	Senior Statistician	TBC	Senior Statistician
**Date**	21st December 2017		
**Protocol Version and Date**	v3.2 (14/07/2016)		

1. Change Control

Protocol version	Updated SAP version no.	Section number changed	Description of change	Date changed
3.2	2.0	18	Further detail on how withdrawls from study will be handled in primary analysis.	04/10/2017
3.2	2.0	17.2	Categories for age added that are required for Eudract reporting	04/10/2017
3.2	2.0	17.1 & 20.1	The word 'randomisation' replaced with 'registration'	04/10/2017
3.2	2.0	20.2	Clarification of how AE/SAE data to be presented	04/10/2017

2. Approval and agreement

SAP Version Number being approved:___________________________

Trial Statistician

Name ____

Signed _________________________________ Date ___________

Senior Statistician

Name ____

Signed _________________________________ Date ___________

Chief Investigator/clinical lead

Name ____

Signed _________________________________ Date ___________

OR Electronic approval attached ☐

Chief Investigator/clinical lead

Name ____

Signed _________________________________ Date ___________

OR Electronic approval attached ☐

3. Roles and responsibilities

Andrew McKay (Department of Biostatistics, University of Liverpool): Trial Statistician

Dr Ashley Jones (Department of Biostatistics, University of Liverpool): Supervising Statistician

Professor Athimalaipet V Ramanan (University Hospitals Bristol NHS Foundation Trust): Co-Chief Investigator

Professor Michael W Beresford (Alder Hey Children's NHS Foundation Trust): Co-Chief Investigator

Professor John Whitehead (Lancaster University): Independent statistical review of the primary outcome section

Author's contributions

A.Jones proposed the statistical analysis plan. A.McKay provided comment on drafts. J.Whitehead provided his expertise to comment on the primary outcome section. A.McKay, A.Jones, A.Ramanan and M.Beresford read, amended and approved the statistical analysis plan.

4. List of abbreviations and definitions of terms

AC	Anterior-Chamber
ACR	American College of Rheumatology
AE	Adverse Event
AESI	Adverse Events of Special Interest
ALT	Alanine Aminotransferase
ANA	Anti-Nuclear Antibody
APTITUDE	A phase II trial of Tocilizumab in anti-TNF refractory patients with JIA associated uveitis
AST	Aspartate Aminotransferase
CHAQ	Childhood Health Assessment Questionnaire
CHQ	Childhood Health Questionnaire
CI	Confidence Interval
CMO	Cystoid Macular Oedema
CONSORT	Consolidated Standards of Reporting Trials
CRF	Case Report Form
CRP	C-Reactive Protein
CTRC	Clinical Trials Research Centre
CTU	Clinical Trials Unit
DMARD	Disease Modifying Anti-Rheumatic Drugs
DsDNA	Deoxyribonucleic Acid
EM	Expectation-Maximization
ENA	Extractable Nuclear Antigens
ESR	Erythrocyte Sedimentation Rate
GCP	Good Clinical Practice
HDL	High-Density Lipoprotein
Hg	Hectogram
IDSMC	Independent Data and Safety and Monitoring Committee
ILAR	International League of Associations for Rheumatology
IMP	Investigational Medicinal Product
IOP	Intraocular Pressure
IQR	Inter-Quartile Range
ITT	Intention to Treat
IV	Intravenous
JADAS	Juvenile Arthritis Disease Activity Score
JIA	Juvenile Idiopathic Arthritis
kg	Kilogram
LDL	Low-Density Lipoprotein
LOCS III	Lens Opacities Classification System III
LLT	Lower Level Term
LTBI	Latent Tuberculosis Infection
MCRN	Medicines for Children Research Network

MedDRA	Medical Dictionary for Regulatory Activities
MMR	Measles, Mumps and Rubella
MMRV	Measles, Mumps and Rubella Vaccine
MO	Macular Oedema
MTX	Methotrexate
OCT	Optical Coherence Tomography
PDF	Portable Document Format
pH	potential of Hydrogen
PI	Principal Investigator
PP	Per-Protocol
PPD	Purified Protein Derivative
PT	Preferred Term
QC	Quality Control
RCT	Randomised Controlled Trial
RF	Rheumatoid Factor
SAE	Serious Adverse Event
SAP	Statistical Analysis Plan
S/C MTX	Subcutaneous Methotrexate
SD	Standard Deviation
SOC	System Organ Class
SOP	Standard Operating Procedure
SUN	Standardisation of the Uveitis Nomenclature
SUSAR	Suspected Unexpected Serious Adverse Reactions
TB	Tuberculosis
TMG	Trial Management Group
TNF	Tumor Necrosis Factor
TSC	Trial Steering Committee
ULN	Upper Limit of Normal
VEGF	Vascular Endothelial Growth Factor
WBC	White Blood Cell

Contents

1. Change Control ... 1
2. Approval and agreement ... 2
3. Roles and responsibilities ... 3
4. List of abbreviations and definitions of terms ... 4
5. Statement of Compliance ... 8
6. Background and Rationale ... 9
7. APTITUDE Study Objectives ... 9
8. Investigational Plan and Study Design ... 9
8.1 Overall study design and plan description ... 9
8.2 Treatments studied ... 10
8.3 Treatment compliance ... 10
8.4 Patient population studied ... 10
8.4.1 Inclusion criteria ... 10
8.4.2 Exclusion criteria ... 11
8.5 Removal of patients from therapy or assessment ... 14
8.6 Consent process ... 14
8.7 Blinding ... 15
8.8 Method of assignment to treatment ... 15
8.9 Sequence and duration of all study periods, ... 16
8.10 Schedule of assessments ... 17
9 Listing of Outcomes ... 18
9.1 Primary outcome(s) ... 18
9.2 Secondary outcomes ... 18
10 Determination of Sample Size ... 20
11 Study Framework ... 20
12 Confidence Intervals, p-values and Multiplicity ... 20
13 Timing and Objectives of Interim and Final Analyses ... 21
13.1 Interim monitoring and analyses ... 21
13.2 Final analysis ... 21
14 Disposition of Participants ... 21
14.1 Screening, eligibility and recruitment ... 21
14.2 Post registration discontinuations ... 23
15 Protocol Deviations ... 23
16 Unblinding ... 24
17 Efficacy Evaluations ... 24
17.1 Data Sets Analysed ... 24
17.2 Demographic and Other Baseline Characteristics ... 25
17.3 Compliance with treatment ... 27
17.4 Analysis of outcomes ... 27
17.4.1 Primary Outcome ... 27
17.4.2 Safety, tolerability and compliance ... 29
17.4.3 Use of Corticosteroids over duration of study period and throughout follow up 32
17.4.4 Optic and Ocular ... 37
17.4.5 Quality of Life assessment (CHQ and CHAQ) ... 43
17.4.6 American College of Rheumatology (ACR) Pedi core set criteria: at ACR30, ACR50, ACR70, ACR90 and ACR100 levels ... 46

17.4.7 Number of participants requiring change in biologic /disease-modifying anti-rheumatic drugs (DMARDs) therapy due to disease flare of their arthritis or failure to respond to treatment of arthritis ... 48
17.4.8 Number of participants undergoing flare of arthritis ... 48
17.4.9 Number of participants in remission on and off medication of their JIA ... 49
17.4.10 Juvenile Arthritis Disease Activity Score (JADAS) ... 52
17.4.11 Number of participants with minimum disease activity ... 54
18 Missing data and withdrawals ... 57
19 Additional analyses ... 58
20 Safety Evaluations ... 59
20.1 Data sets analysed ... 59
20.2 Presentation of the data ... 59
21 References ... 63

5. Statement of Compliance

This Statistical Analysis Plan (SAP) provides a detailed and comprehensive description of the pre-planned final analyses for the study “APTITUDE”. The planned statistical analyses described within this document are compliant with those specified in brief within the APTITUDE protocol version 3.2 (14/07/2016).

This SAP comprehensively describes the planned final analyses.

This study is carried out in accordance with the World Medical Association Declaration of Helsinki (1964) and the Tokyo (1975), Venice (1983), Hong Kong (1989) and South Africa (1996) amendments and will be conducted in compliance with the protocol, Clinical Trials Research Centre (CTRC) Clinical Trials Unit (CTU) Standard Operating Procedures (SOPs) and EU Directive 2001/20/EC, transposed into UK law as the UK Statutory Instrument 2004 No 1031: Medicines for Human Use (Clinical Trials) Regulations 2004.

These planned analyses will be performed by the trial statistician.

This study is a clinical trial of a medicinal product and is registered on the EudraCT database. The statistical analysis plan has been developed to support the posting of results on the EudraCT system. This is a regulatory requirement which should be fulfilled within 12 months after the end of the study as defined within the clinical trial protocol.

The results of the final analysis described within this statistical analysis plan will be contained within a statistical analysis report. This report will be used as the basis of the primary research publications according to the study publication plan.

All analyses are performed with standard statistical software (SAS version 9.3 or later). The finalised analysis datasets, programs and outputs will be archived following Good Clinical Practice guidelines and SOP TM021 Archiving procedure in CTRC. The testing and validation of the statistical analysis programs will be performed following SOP ST001.

6. Background and Rationale

The efficacy of tocilizumab in uveitis and ophthalmology outcomes has not been studied. However, the rationale for anti-IL-6 therapy is strong. Hence the need for a phase II study to give early indications of the clinical effectiveness of Tocilizumab in combination with Methotrexate (MTX) and to decide whether further research is justified.

7. APTITUDE Study Objectives

The primary objective of this trial is to estimate the response rate to Tocilizumab in combination with MTX in children with Juvenile Idiopathic Arthritis (JIA)-associated uveitis who have already failed anti-TNF therapy, and to determine whether further research into the use of this intervention for the treatment of anti-TNF refractory JIA-associated uveitis should be conducted.

To conduct a preliminary evaluation of the short term safety and tolerability of Tocilizumab in combination with MTX in children with JIA-associated uveitis, with regards to ocular complications of treatment, adverse events and laboratory assessments.

To assess the efficacy of treatment with Tocilizumab to permit concomitant medication reduction, in particular topical and parenteral steroids.

To develop a fully consented, trial-related Bio Bank for subsequent investigation.

8. Investigational Plan and Study Design

8.1 Overall study design and plan description

The trial will be conducted following a two-stage Simon design (Simon, 1989). After 10 participants have completed their three month follow up there will be an interim analysis. If there are 8 or more failures out of these 10 then the trial will stop with the conclusion that the study of Tocilizumab should be abandoned. If there are fewer than 8 failures then the study will continue until a further 12 participants have received treatment, giving a total

sample size of 22. If amongst these 22 participants there are 15 or more failures then it will be concluded that the further study of Tocilizumab should be abandoned. If further study of the drug is not abandoned at either the interim of the final analysis, then a recommendation to conduct a comparative, randomised phase III trial will be made.

8.2 Treatments studied

All participants will receive injections every 2 or 3 weeks of Tocilizumab alongside MTX treatment. The dosage will be calculated based on patient body weight.

8.3 Treatment compliance

The participant or the parent/guardian of a participant will maintain a diary for all trial medication.

8.4 Patient population studied

8.4.1 Inclusion criteria

1) Children and young people aged ≥ 2 and <18 years.

2) At the time of trial screening the participant must have active anterior uveitis, defined as "2 readings of cellular infiltrate in anterior chamber of SUN criteria grade ≥1+ or more during the preceding 6 weeks, the latest reading must be at the time of screening".

3) Participants must have failed MTX (minimum dose of 10mg/m^2, with a maximum dose of 20mg/m^2 and not to exceed 25mg/participant). The participant must have been on MTX for at least 12 weeks and have been on a stable dose of MTX for 4 weeks prior to screening visit.

4) Participants must have failed an anti TNF agent and have been on at least one anti-TNF agent regardless of dose for at least 12 weeks at any time previously.
   If a participant has received previous treatment with any of the following biologic agents, these must have been discontinued according to the following timelines prior to registration:

   Infliximab 8 weeks prior to registration.
   Etanercept 2 weeks prior to registration.

Adalimumab 4 weeks prior to registration.

Abatacept 8 weeks prior to registration.

Canakinumab 20 weeks prior to registration.

Rilonacept 6 weeks prior to registration.

Anakinra 1 week prior to registration.

5) Written informed consent of participant or parent/legal guardian, and assent where appropriate.

6) Participant and parent/legal guardian willing and able to comply with protocol requirements.

7) For participants of reproductive potential (males and females), use of a reliable means of contraception throughout their trial participation (abstinence is an acceptable method of contraception as long as this is the usual and preferred lifestyle of the patient).

8) Post pubertal females must have a negative serum pregnancy test within 14 days prior to registration.

9) Able to commence trial treatment within 2 weeks of the screening visit.

8.4.2 Exclusion criteria

1) Uveitis without a diagnosis of JIA fulfilling ILAR diagnostic criteria for JIA (all subgroups that have uveitis).

2) Currently on Tocilizumab or has previously received Tocilizumab.

3) Previous registration into the APTITUDE trial.

4) Participation in another clinical trial of investigational medicinal product within the last 4 weeks or 5 serum half-lives (whichever is longer) prior to registration.

5) More than 6 topical steroid eye drops per day per eye at time of registration (dose must be stable for 1 week prior to registration).

6) For patients on Prednisone or Prednisone equivalent, change of dose within 4 weeks prior to registration.

7) Patients on Prednisone or Prednisone equivalent with a dose >0.2mg/kg per day.

8) No intraocular injection of disease modification agents including steroids and anti-VEGF within 4 weeks prior to registration.

9) No intraocular surgery for previous 12 weeks prior to registration or expected/panned for duration of study.

10) Lack of recovery from recent surgery or surgery within 6 weeks at the time of registration.

11) Intra-ocular pressure ≥ 25mm Hg at time of registration.

12) Patients requiring systemic therapy with oral anti-glaucoma medication.

13) No disease modifying immunosuppressive drugs, other than MTX in the 4 weeks prior to registration.

14) History of active tuberculosis of less than 24 weeks treatment.

15) Latent TB not successfully treated for at least 4 weeks prior to registration (a test for latent tuberculosis infection (LTBI) must be performed within 12 weeks prior to registration).

16) Auto-immune, rheumatic disease or overlap syndrome other than JIA.

17) Females who are pregnant, lactating, or intending to become pregnant during trial

18) Known human immunodeficiency virus infection or other condition characterized by a compromised immune system.

19) Any history of alcohol or drug abuse within 24 weeks prior to registration.

20) Any active acute, sub-acute, chronic, or recurrent bacterial, viral, systemic fungal, infection or any major episode of infection requiring hospitalisation or treatment with IV antibiotics within 4 weeks of registration or treatment with oral antibiotics within 2 weeks of registration.

21) History of reactivation or new onset of a systemic infection such as herpes zoster or Epstein−Barr virus within 8 weeks prior to registration.

22) Hepatitis B surface antigen or hepatitis C antibody positivity or chronic viral or autoimmune hepatitis.

23) History of concurrent serious gastrointestinal disorders.

24) Evidence of current serious uncontrolled concomitant cardiovascular (including hyperlipidemia), nervous system, pulmonary (including obstructive pulmonary disease), renal and hepatic disease.

25) History of or current cancer or lymphoma.

26) Persistently poorly controlled severe hypertension (>95th percentile for height / age).

27) Uncontrolled diabetes mellitus.

28) History of severe allergic or anaphylactic reactions to human, humanized or murine monoclonal antibodies.

29) No live attenuated vaccines (including seasonal nasal flu vaccine, varicella vaccine for shingles or chickenpox, MMR or MMRV, oral polio vaccine and vaccines for yellow fever, measles, mumps or rubella) 4 weeks prior to registration, throughout the duration of the trial and for 8 weeks following the last dose of study drug.

30) Previous treatment with any cell-depleting therapies, including investigational agents or approved therapies (e.g. CAMPATH, anti-CD4, anti-CD5, anti- CD3, anti-CD19 and anti-CD20).

31) Treatment with intravenous gamma globulin or plasmapheresis within 24 weeks of registration.

32) Any previous treatment with alkylating agents such as chlorambucil, or with total lymphoid irradiation.

33) Any significant medical or surgical condition that would risk the patient's safety or their ability to complete the trial.

34) Any joint injections within 4 weeks prior to registration.

35) Any psychological condition that in the opinion of the principal investigator would interfere with safe completion of the trial.

36) Demonstrations of clinically significant deviations from the following laboratory parameters:

    Serum creatinine > 1.5 × the upper limit of normal (ULN) for age and sex.
    AST or ALT > 1.5 × the ULN for age and sex.
    Total bilirubin > 1.3 mg/dL (>23 µmol/L).
    Platelet count < 150 × 103/µL (< 150,000/mm3).
    Hemoglobin < 7.0 g/dL (< 4.3 mmol/L).
    White blood cell (WBC) count < 5,000/mm3 (< 5.0 × 109/L).
    Neutrophil count < 2,500/mm3 (< 2.5 × 109/L).

8.5 Removal of patients from therapy or assessment

Patients who miss two consecutive doses or three doses in total (whichever is first) of Tocilizumab injection should cease trial treatment and will be recorded as a withdrawal from treatment.

8.6 Consent process

Informed consent is a process initiated prior to an individual agreeing to participate in a trial and continues throughout the individual's participation. Written informed consent is required for all trial participants. In obtaining and documenting informed consent, the

investigator should comply with applicable regulatory requirements and should adhere to good clinical practice and to the ethical principles that have their origin in the Declaration of Helsinki.

8.7 Blinding

This is a single arm trial, therefore this section is not relevant.

8.8 Method of assignment to treatment

This is a single arm trial and therefore there is no randomisation involved. Patients are registered using a secure (24-hour) web based registration programme. Participant registration number will be displayed on a secure webpage following confirmation of eligibility.

8.9 Sequence and duration of all study periods,

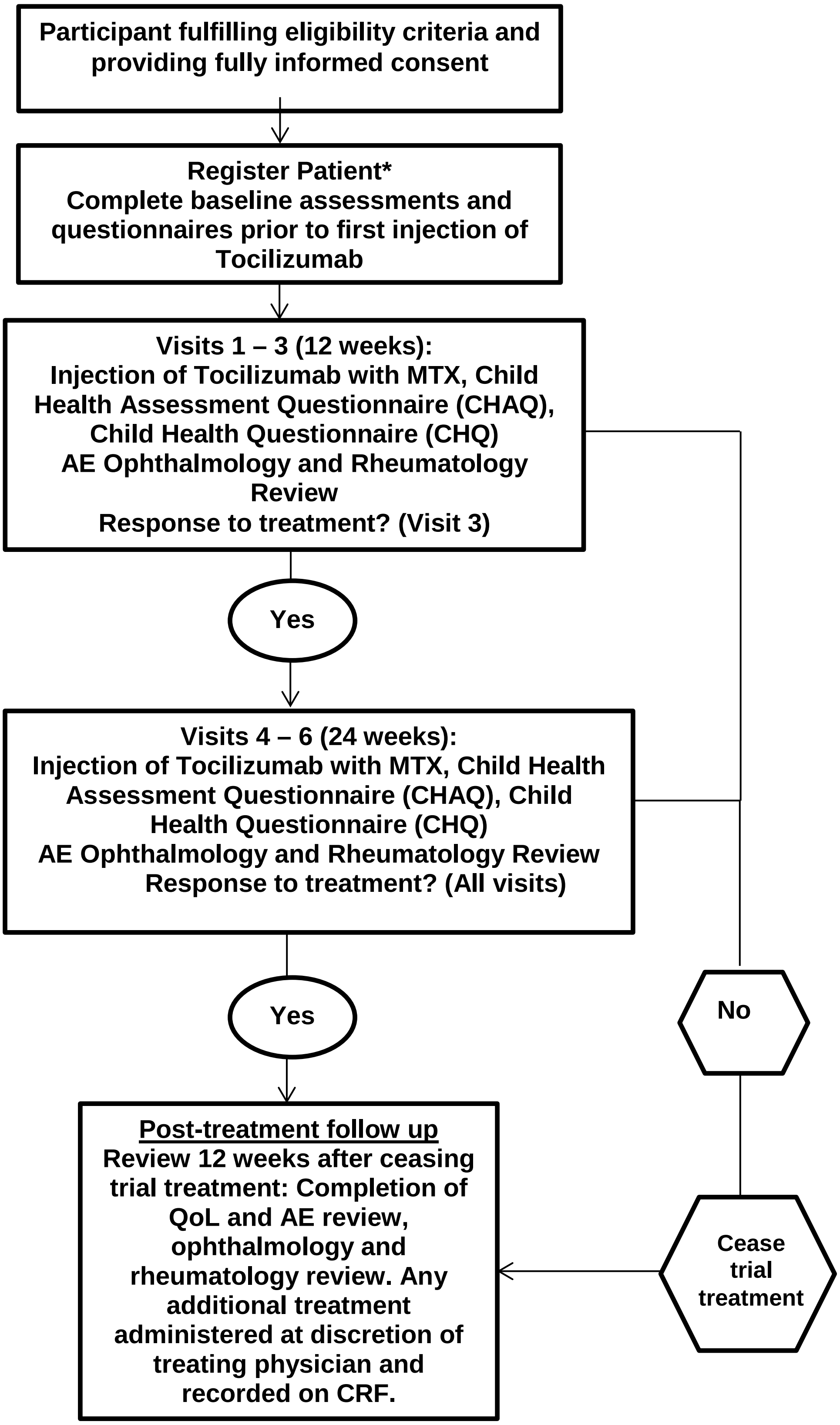

8.10 Schedule of assessments

**Assessment (Procedure/ Activity)**	**Screening**	**Baseline**	**Visit 1**	**Visit 2**	**Visit 3**	**Visit 4**	**Visit 5**	**Visit 6**	**Visit 7**
**Weeks**		0 (-7/+7 days)	4 (-7/+7 days)	8 (-7/+7 days	12 (-7/+7 days)	16 -7/+7 days)	20 (-7/+7 days)	24 (-7/+7 days)	36 (-7/+7 days)
	Screening	Baseline/ Registration	Study Visit	Study Visit	Assessment of endpoints	Study Visit	Study Visit	End of treatment	End of trial
Written and informed consent	X								
Confirm consent	X	X	X	X	X	X	X	X	X
Assessment of eligibility criteria	X	X							
Review of Medical/ Ophthalmic/ Surgical History	X								
Review of concomitant medications	X	X	X	X	X	X	X	X	X
Pregnancy test	X		X	X	X	X	X	X	X
Purified protein derivative Tuberculin Skin Test/ Test latent TB as locally performed	X								
Urinalysis	X	X	X	X	X	X	X	X	X
Study intervention (Injection)		X	X	X	X	X	X	X	
Compliance with study intervention		X	X	X	X	X	X	X	
Physical Examination		X	X	X	X	X	X	X	X
Vital signs (heart and respiratory rate and blood pressure)	X		X	X	X	X	X	X	X
Height/ Weight	X	X	X	X	X	X	X	X	X
Dispense treatment diary		X	X	X	X	X	X	X	
CHQ		X	X	X	X	X	X	X	X
CHAQ		X	X	X	X	X	X	X	X
Haematological analysis	X	X*	X	X	X	X	X	X	X
Biochemical analysis	X	X*	X	X	X	X	X	X	X
ANA dsDNA ENA		X						X	
Samples for Biobank		X			X				X
Vision Assessments[#]	X	X	X	X	X	X	X	X	X
Optical coherence tomography	X		X	X	X	X	X	X	X
AC cells and flare assessment[#]	X	X	X	X	X	X	X	X	X
BIO Score	X		X	X	X	X	X	X	X
Cataract scoring	X		X	X	X	X	X	X	X
Goldman tonometry or tonopen	X		X	X	X	X	X	X	X
Standard ACR Pedi Score Set Outcome Variables		X	X	X	X	X	X	X	X
Tanner Score		X			X			X	X
Assessments of Adverse and Serious Adverse Events		X	X	X	X	X	X	X	X

NB Registration should take place no later than 2 weeks after the beginning of screening

9 Listing of Outcomes

9.1 Primary outcome(s)

The primary endpoint is response to treatment*.

** Response to treatment is defined as per SUN criteria as a 2 step decrease in the level of inflammation (anterior chamber cells) or decrease to zero between baseline (prior to trial treatment initiation) and after 12 weeks of treatment.*

9.2 Secondary outcomes

1) Safety, tolerability and compliance
   a. Adverse events (AEs), serious adverse events (SAEs) and Adverse Events of Special Interest (AESI)
   b. Laboratory parameters (haematological and biochemical analysis and urinalysis)
   c. Participant diaries and dosing records will determine tolerability and compliance throughout the trial treatment period

2) Use of Corticosteroids over duration of study period and throughout follow up, including:
   a. Total oral corticosteroid dose
   b. Reduction in and rate of systemic corticosteroid dose from entry dose
   c. Topical corticosteroid use (frequency) compared to usage at registration.

3) Optic and Ocular
   a. Visual acuity measured by Age-appropriate LogMAR assessment
   b. Number of participants with resolution of associated optic nerve or macular oedema (as assessed by slit lamp biomicroscopy or optical coherence tomography (OCT)).
   c. Number of patients who are able to reduce topical or systemic agents for ocular hypertension.

d. Number of participants with disease control (defined as zero cells, with topical treatment at 12 weeks treatment visit and 24 weeks treatment visit.)
e. Number of participants entering disease remission (defined as zero cells, without topical treatment at 12 and 24 weeks treatment visit)
f. Duration of sustaining inactive disease (zero cells, with or without topical treatment.)
g. Failure to reduce eye drops to 2 drops/day by or at the 12 weeks visit

4) Quality of Life assessment (Childhood Health Questionnaire (CHQ), Childhood Health Assessment Questionnaire (CHAQ))

5) American College of Rheumatology (ACR) Pedi core set criteria: at ACR30, ACR50, ACR70, ACR90 and ACR100 levels

6) Number participants requiring change in biologic / Disease-modifying anti-rheumatic drugs (DMARDs) therapy due to disease flare of their arthritis or failure to respond to treatment for their arthritis.

7) Number of participants undergoing flare of arthritis, in remissions on and off medication of their JIA and with minimum disease activity.

8) Participants score of the Juvenile Arthritis Disease Activity Score (JADAS). The JADAS comprises four components: (1) physician global assessment of disease activity (2) parent/patient global assessment of well-being (3) active joint count, in 27, 71 or 10 joints; and (4) erythrocyte sedimentation rate (ESR).

10 Determination of Sample Size

The trial will be conducted following a two-stage Simon design (Simon, 1989). After 10 participants have completed their three month follow up there will be an interim analysis. If there are 8 or more failures out of these 10 then the trial will stop with the conclusion that the study of Tocilizumab should be abandoned. If there are fewer than 8 failures then the study will continue until a further 12 participants have received treatment, giving a total sample size of 22. If amongst these 22 participants there are 15 or more failures then it will be concluded that the further study of Tocilizumab should be abandoned. If further study of the drug is not abandoned at either the interim of the final analysis, then a recommendation to conduct a comparative, randomised phase III trial will be made.

The null hypothesis (response=20%) reflects a response rate of no clinical benefit while the alternative hypothesis (response=50%) reflects a desired response. The interim and final sample sizes and the critical values for abandoning Tocilizumab at each stage have been chosen to achieve the following properties. If the true success probability is 0.2 then we will recommend further study of Tocilizumab with probability less than 0.05 (falsely pursuing a non-promising therapy). If the true success probability is 0.5 then we will recommend further study of Tocilizumab with probability greater than 0.9 (correctly pursuing a promising therapy).

11 Study Framework

The overall objective for each of the secondary outcomes is to test the efficacy of tocilizumab in combination with MTX.

12 Confidence Intervals, p-values and Multiplicity

See analysis section of 17.4.1 for details.

13 Timing and Objectives of Interim and Final Analyses

13.1 Interim monitoring and analyses

After 10 participants have completed their three month follow up there will be an interim analysis. If there are 8 or more failures out of these 10 then the trial will stop with the conclusion that the study of Tocilizumab should be abandoned. If there are fewer than 8 failures then the study will continue until a further 12 participants have received treatment, giving a total sample size of 22.

The results of the point estimates and confidence intervals for the probability of success will be computed using the exact method described in Jovic and Whitehead (2010).

13.2 Final analysis

The end of the trial will be considered as the date of the final database lock.

14 Disposition of Participants

14.1 Screening, eligibility and recruitment

Screening logs will be summarised by site in a table detailing:

i) the number of patients who were assessed for eligibility at the screening visit,

ii) those who met the study inclusion criteria at screening (expressed as a frequency and a % with the denominator being i),

iii) those who did not meet the study inclusion criteria at screening (expressed as a frequency and a % with the denominator being i),

iv) those who were eligible at screening and consent obtained, (expressed as a frequency and a % with the denominator being ii),

v) those who were eligible at screening and consent not obtained, (expressed as a frequency and a % with the denominator being ii),

vi) those who provided consent but were not registered (expressed as a frequency and a % with the denominator being iv),

vii) those who provided consent and were registered (expressed as a frequency and a % with the denominator being iv).

Reasons for ineligibility will be summarised by site and overall in a table.

Frequencies will be presented along with percentages using the denominator as iii).

Reasons for consent declined will be summarised by site and overall in a table with the following categories:

1. Does not want to consent (unwilling to provide reason)
2. Unwilling/unable to comply with study requirements
3. Does not wish to consent for other reason (specify).

Frequencies will be presented along with percentages using the denominator as v).

A CONSORT flow diagram (CONSORT, 2010) will be used to summarise the number of patients who were:

- assessed for eligibility at screening
    - eligible at screening
    - ineligible at screening*
- eligible and registered
- eligible but not registered*
- received the allocation
- did not receive the allocation*
- lost to follow-up*
- discontinued the intervention*
- registered and included in the primary analysis
- registered and excluded from the primary analysis*

*reasons will be provided.

A recruitment summary table will be presented showing the following for each centre: centre code, hospital name, dates site opened/closed to recruitment, dates of first/last registration and total number registered.

14.2 Post registration discontinuations

Every effort will be made to collect follow-up data even if participants have withdrawn from trial intervention. In some cases it may not be possible to continue follow-up of trial participants due to transfer to a non-participating centre, loss to follow-up etc. in which case the withdrawal form will be completed.

Withdrawals from treatment and/or trial will be presented as line listings detailing:

- Registration number
- Site
- Date of registration
- Date of discontinuation
- Who made the decision to withdraw participant from trial:
    - Clinician
    - Participant/parent/guardian
    - Clinician and participant
- Reason for discontinuation:
    - Death
    - Lost to follow-up
    - Transfer to a non-participating centre
    - Withdrawal of consent for follow-up
    - Other reason (specify)
- Level of withdrawal (from treatment and/or trial).

15 Protocol Deviations

Protocol deviations that will be reported are defined in the monitoring plan v1.0 19/08/2016 for the trial. All protocol deviations of the listed protocol specifications have been included in the trial monitoring reports presented overall and by site when numbers recruited per site are sufficient. Protocol deviations are classified prior to unblinding of treatment. All protocol deviations will be defined and signed-off using ST001TEM03 Protocol deviations and population exclusions template prior to unblinding. The number (and percentage) of patients with each separate protocol deviation will be presented in this analysis report along with the number (and percentage) of patients with (i) at least one

major protocol deviation; (ii) at least one minor protocol deviation; and (iii) at least one protocol deviation of any classification (minor or major). These will also be summarised across site. No formal statistical testing will be undertaken. Line listings will be presented where appropriate.

16 Unblinding

Due to the nature of the study there is no blinding possible.

17 Efficacy Evaluations

All results summaries will be presented to 1 decimal place except for the primary outcome analysis results (median unbiased estimator, 95% confidence interval and p-value) which will be presented to 3 decimal places.

17.1 Data Sets Analysed

The principle of intention-to-treat (ITT), as far as is practically possible, will be the main strategy of the analysis adopted for primary and secondary outcomes. Patients that withdrew consent for trial continuation will contribute outcome data up until the point of withdrawal unless the patients' parents/guardians specifically request that the data are not to be used.

The membership of the analysis set for each outcome will be determined and documented and reasons for participant exclusion will be given. Reasons may include missing data, loss to follow up and treatment withdrawal.

Patients to be excluded from the primary and secondary analysis population will be defined in template 'ST001TEM03 Protocol deviations and population exclusions template'.

For efficacy outcomes, any dates that have the month (mm) and year (yyyy) recorded but not the specific day will be imputed as the start of the month (01/mm/yyyy). Any dates that have the year (yyyy) recorded but not the specific day or month will be imputed as the start of the year (01/01/yyyy).

For partial dates on the concomitant medication forms, any start dates that have the month (mm) and year (yyyy) recorded but not the specific day will be imputed as the start of the month (01/mm/yyyy)*. Any end dates that have the month (mm) and year (yyyy) recorded but not the specific day will be imputed as the final day of the month**. Any start dates that have the year (yyyy) recorded but not the specific day or month will be imputed as the start of the year (01/01/yyyy)*. Any end dates that have the year (yyyy) recorded but not the specific day or month will be imputed as the end of the year (31/12/yyyy)**.

* If an imputed date proceeds the registration date take the registration date.
* If an imputed date exceeds the last treatment visit date take the last treatment visit date.

17.2 Demographic and Other Baseline Characteristics

Patients will be described with respect to the following:

- *Demographics*: Age[#&$10], gender[$1],weight (<30kg, ≥30kg)[#]
- *Number of study eyes*[$2]
- *Physician global assessment of disease activity*[#]
- *Auto anti-body screen*: Anti-nuclear antibody (ANA)[$3], Double-stranded deoxyribonucleic acid (DsDNA)[$3], Extractable nuclear antigen (ENA)[$3]
- *Type of JIA:* ILAR classification[$4]
- *JIA disease duration*[#]
- *Joint counts**: Active[#], swollen[#]
- *Vision assessment*: LogMAR score[#] *[in study eye(s)]*
- *Slit lamp examination [in study eye(s)]*:
    - *Test*: AC cells (SUN)[$5], flare score (SUN)[$5],
    - *LOCS III Grading*: pseudophakic[$6], nuclear[$7], cortical[$8], posterior[$9]
    - *Other Structural Changes*: central band-keratopathy – covering visual axis[$6], synchiae[$6], iris bombe[$6], membrane formation[$6], neovascularisation[$6]
- *Intraocular pressure*[#] *[in study eye(s)]*
- *Number of topical steroid drops taken per day*.

* If 'Not done' has been selected for a joint count assessment treat as no joints are active/swollen (as directed by the Chief Investigators).

Continuous

$1 Categorical: Male/Female

$2 Categorical: Unilateral/Bilateral

$3 Categorical: Positive/Negative/Not Done

$4 Categorical: Systemic arthritis, Persistent oligoarthritis, Extended oligoarthritis, Polyarthritis RF positive, Polyarthritis RF negative, Psoriatic arthritis, Enthesitis-related arthritis, Undifferentiated arthritis

$5 Categorical: 0/0.5+/1+/2+/3+/4+

$6 Categorical: Yes/No

$7 Categorical: N0/NI/NII/NIII

$8 Categorical: Ctr/CI/CII/CIII/CIV/Aphakic or Pseudophakic/No Cortical Cataract

$9 Categorical: O/PI/PII/PIII

$10 Categorial: Eudract defined age-groups: In Utero, preterm newborn-gestational age <37 weeks, newborns (0-27 days), infants and toddlers (28 days-23 months), children (2-11 years), adolescents (12-17 years), between 18-64 years, >= 65 years

Note: Reasons for any tests not being done will be summarised if provided.

Categorical data will be summarised by numbers and percentages. Continuous data will be summarised by mean, SD, median, IQR (upper and lower quartile) and range (maximum and minimum values) regardless of whether the data are skewed or not. A summary table will be presented overall and separate summary tables for each site. The characteristics highlighted above with "*[in study eye(s)]*" will be presented in three ways: (i) Eye-level; (ii) Best-case; (iii) Worst-case.

Tests of statistical significance will not be undertaken for baseline characteristics; rather the clinical importance of any imbalance will be noted.

17.3 Compliance with treatment

The participant or the parent/guardian of a participant will maintain a diary for all trial and other medications that are administered outside of the trial visit (i.e. at home).

For *trial allocated treatment* the diary will collect information on:

(i) Vial number

(ii) Time/ date of administration

(iii) Volume/dose

(iv) Any problems with administration/ protocol adherence

CRF "Treatment diary" shows the number of injections given for each participant. These are used to determine compliance with the trial treatment. The number of suspected missing doses is the difference between the expected number of doses and CRF "Accountability Log" shows the number of vials issued and the number returned (both used and unused).

No formal analysis will be undertaken for this outcome. Summary statistics will be presented as line listings per patient by treatment including information of expected number of IMP doses, actual number of IMP doses, number of suspected missing doses, number of vials issued, number of vials returned used and number of vials returned unused.

17.4 Analysis of outcomes

17.4.1 Primary Outcome

The primary endpoint is response to treatment*.

** Response to treatment is defined as per SUN criteria as a 2 step decrease in the level of inflammation (anterior chamber cells) or decrease to zero between baseline (prior to trial treatment initiation) and after 12 weeks of treatment.*

Derivation

This can be found on subsection "Subform Binocular indirect opthalmoscope and Slit lamp exam" of the treatment visit CRF under "AC Cells (SUN)" for the right and left eye.

Analysis

The parameter representing the effectiveness of the experimental treatment will be the probability that a study patient succeeds.

The analysis methods described below have been taken from the approach described in Jovic and Whitehead for the analysis of two stage phase II trials (Jovic and Whitehead, 2010).

The null hypothesis is $H_0: p = p_0$ where p represents the probability that a patient responds and represents the effectiveness of Tocilizumab , p_0 is equal to the probability of success (response to treatment) of standard therapy was made by a judgement of the investigators of this trial. The alternative hypothesis is $H_1: p = p_1$, where $p_1$would be the probability of success of Tocilizumab which would be clinically relevant and prompt further investigation.
In this study:

$$H_0: p = 0.2$$
$$H_1: p = 0.5$$

A two stage design has five quantities of interest $(l_1, u_1, u_2, n_1, n_2)$

Where:

l_1 is equal to the maximum number of successes that would lead to the study ending after stage 1 and the conclusion that no further investigation is warranted.

u_1 is equal to the minimum number of success that would lead the study to stop after stage 1 and the conclusion that further investigation is warranted.

u_2 is equal to the minimum number of successes required at the end of stage 2 to recommend further investigation

n_1 is equal to the total number of patients at the end of stage 1

n_2 is equal to the total number of patients at the end of stage 2

The design used for the APTITUDE trial uses a Simon design which is a special case of the two stage design with $u_1 = \infty$, meaning that stopping to reject H_0 at the end of stage 1 is not possible.

The four quantities of interest in APTITUDE are therefore: (2, 14,10,22).

$P(p)$ is the probability of obtaining evidence in a repeat trial of the same design that supports H_1 as strongly or more strongly than that observed, if in fact H_0 is true;

and

$Q(p)$ is the probability of obtaining evidence in a repeat trial of the same design that supports H_1 more strongly than that observed, if in fact H_0 is true.

S_1 is the number of successes amongst the first n_1 patients.

S_2 is the number of successes amongst the total n_2 patients.

The functions $P(p)$ and $Q(p)$ are computed using the binomial distribution and are defined as follows:

$$P(p) = \begin{cases} P(\{S_1 > l_1 \text{ and } S_2 \geq s_2\}|p) \text{ if } s_1 > l_1 \\ 1 - P(S_1 < s_1|p) \text{ if } s_1 \leq l_1 \end{cases}$$

$$Q(p) = \begin{cases} P(\{S_1 > l_1 \text{ and } S_2 > s_2\}|p) \text{ if } s_1 > l_1 \\ 1 - P(S_1 \leq s_1|p) \text{ if } s_1 \leq l_1 \end{cases}$$

The approximate median unbiased estimate of p will be calculated by the average of P_M^- and P_M^+, where $P(P_M^-) = Q(P_M^+) = 0.5$.

The confidence interval (p_L, p_U) will be estimated using exact methods as per the (Clopper and Pearson, 1934) approach, where $P(p_L) = 0.025$ and $Q(p_U) = 0.975$.

Line listings for each participant that responded to treatment will be presented detailing: registration number, registration date, eligible eye(s), response eye(s), AC cells scores at baseline and response visit.

17.4.2 Safety, tolerability and compliance

17.4.2.1 Adverse events (AEs), serious adverse events (SAEs) and Adverse Events of Special Interest (AESI)

See Section 20.

17.4.2.2 Laboratory parameters (haematological and biochemical analysis and urinalysis)

Derivation

The laboratory parameters for haematological and biochemical analysis can be found on subsection “Subform Haematological and Biochemical Assessments” of the treatment visit CRF under “Haematological Assessments” and “Biochemical Assessments”. It can also be found here whether the assessment result was “Normal”, “Abnormal” or “Not done”, and also whether the result was “Clinically significant” (only applicable if the result was abnormal).

The parameters to be reported on for haematological assessments are:

- Haematocrit
- Haemoglobin
- Red blood cell count
- White blood cell count
- Neutrophils
- Lymphocytes
- Monocytes
- Basophils
- Eosinophils
- Platelet count
- Erythrocyte sedimentation rate
- Plasma viscosity (only done if ESR not available

The parameters to be reported on for biochemical assessments are:

- C- Reactive protein (CRP)
- Urea
- Creatinine
- Sodium
- Potassium
- Calcium
- Inorganic phosphate
- Glucose
- Chloride
- Bicarbonate
- Total bilirubin
- LDL
- HDL
- Triglycerides
- Total cholesterol
- Alanine aminotransferase (ALT)
- Aspartate aminotransferase (AST)

The laboratory parameters for urinalysis can be found on subsection “Subform Urinalysis Test, Physical Exam and Vital Signs” of the treatment visit case report form (CRF) under “Urinalysis test”. It can also be found here whether the assessment result was “Normal” or “Abnormal”, and also whether the result of Microscopic urinalysis was “Clinically significant” (only applicable if the result was abnormal).

The parameters to be reported on for urinalysis are:

- Protein
- Glucose
- Blood
- Leukocyte esterase
- Specific gravity
- pH

Due to differences in equipment across the sites, some values may have an upper/lower limit rather than an exact value. For when a value has an upper limit (for example ‘<5’), then the value of ‘0’ should be used as this is the most extreme value a recording could be. In the instances when a value has a lower limit (for example ‘>10’) then the lower limit should be used (in this example ‘10’) as it is not practical to use a value of infinity.

Analysis

Mean profile plots and individual plots will be presented for every haematological and biochemical assessment. 1-standard error bars will be displayed for each visit on the mean profile plots. Summary statistics will also be presented in a table for each time point detailing the number of normal/abnormal/not done assessments, and for the abnormal results the frequency and percentage of clinically significant results for each haematological, biochemical and urinalysis assessment.

For each time point the following will be presented: mean, SD, median, IQR, range, and the number of patients with abnormal values. Tables for each time point will detail the mean

change from baseline to each time point for each of the haematological and biochemical assessments.

For urinalysis, a table will be presented for each time point as line listings for each patient detailing the assessments done, whether the result was abnormal/normal and whether the result was clinically significant (yes/no).

17.4.2.3 Participant diaries and dosing records will determine tolerability and compliance throughput the trial treatment period

Derivation

CRF "Treatment diary" shows the number of injections given for each participant. These are used to determine compliance with the trial treatment.

Analysis

No formal analysis will be undertaken for this outcome. Summary statistics will be presented as line listings per patient including information of expected number of IMP doses, actual number of IMP doses, number of suspected missing doses, number of vials issued, number of vials returned used and number of vials returned unused.

17.4.3 Use of Corticosteroids over duration of study period and throughout follow up

17.4.3.1 Total oral corticosteroid dose

Derivation

Oral corticosteroid dose can be found on CRF "Concomitant Medication Form" and are defined as all prednisolone medications (under "Medication (generic name)") given orally (Route code 2).

If the concomitant medication form does not specify which eye a particular dose is for then it is assumed that the medication is for the eligible eye/eyes.

The total dose is calculated by summing the daily doses of each oral treatment on the Concomitant Medication Form from the start date to the end date until the 24 week visit date (or the date that a patient fails to respond or withdraws from the trial). If no end date

is recorded on the Concomitant Medication Form then it will be assumed that the patient is still receiving the treatment until a new oral corticosteroid treatment is recorded.

The total dose should be summed across the treatment arm and standardised to per patient years. This is calculated by dividing the total oral dose by the cumulative years all patients are on treatment for.

Analysis

The incident rate and 95% confidence intervals will be presented based on Poisson regression. The 'PROC GENMOD' command will be used in SAS, specifying the Poisson distribution and using the log link function. Including the 'ILINK' option applies the inverse link function to provide rate estimates. The 'CL' option produces confidence intervals for the rates. If overdispersion is present (i.e. if the mean and variance are not equal), a negative binomial model will be used instead.

17.4.3.2 Reduction in and rate of systemic corticosteroid dose from entry dose

17.4.3.2.1 Reduction in corticosteroid dose from entry dose

Derivation

The analysis set of this outcome is a subset of all patients, as not everyone will be taking systemic corticosteroids at registration. This outcome will have two analyses undertaken: the time to reduction of systemic corticosteroid dose to <5mg/day, and the time to reduction of systemic corticosteroid dose to 0mg/day. Systemic corticosteroids can be found on CRF "Concomitant Medication Form" and are classed as "systemic" on the database. The entry dose should be identified by taking the dose of a systemic corticosteroid entry on the Concomitant Medication Form which crosses the registration date.

If the concomitant medication form does not specify which eye a particular dose is for then it is assumed that the medication is for the eligible eye/eyes.

Analysis

Censoring within this time to event outcome will occur at the time point that the patient does not have a treatment response. This means that a competing risks model is needed, as patients with failing to respond to treatment are informatively censored (at a lower risk than those who have treatment response). Therefore a competing risks model will be used with the two competing events being reduction in systemic corticosteroid dose and failing to respond to treatment.

The competing risks analysis will be performed using the Fine and Gray model. The 'cmprsk' package in R will be used for this analysis. The Cumulative incidence plots will be presented. Median time to failing to respond to treatment will be calculated and presented with 95% confidence intervals for each risk. If the algorithm does not converge these cannot be presented.

17.4.3.2.2 Rate of systemic corticosteroid dose

Derivation

Systemic corticosteroids can be found on CRF "Concomitant Medication Form" and are classed as "systemic" on the database. If the concomitant medication form does not specify which eye a particular dose is for then it is assumed that the medication is for the eligible eye/eyes.

The total dose is calculated by summing the daily doses of each systemic corticosteroid treatment on the Concomitant Medication Form from the start date to the end date until 24 weeks/withdrawal/failure to respond. If no end date is recorded on the Concomitant Medication Form then it will be assumed that the patient is still receiving the treatment until a new systemic corticosteroid treatment is recorded.

The total systemic corticosteroid dose should be summed and standardised to per patient years. This is calculated by dividing the total systemic corticosteroid dose by the cumulative years all patients are on treatment for.

Analysis

The incident rate and 95% confidence intervals will be presented based on Poisson regression. The ‘PROC GENMOD’ command will be used in SAS, specifying the Poisson distribution and using the log link function. Including the ‘ILINK’ option applies the inverse link function to provide the rate estimate. The ‘CL’ option produces a confidence interval for the rate. If overdispersion is present (i.e. if the mean and variance are not equal), a negative binomial model will be used instead.

17.4.3.2.3 Topical corticosteroid use (frequency) compared to usage at registration

Derivation

This outcome is the time to reduction to ≤1 and 0 drops drop for those patients already on >1 and >0 drops at registration respectively. Topical corticosteroids can be found on CRF “Concomitant Medication Form” and are defined as: prednisolone, dexamethasone, betamethasone, fluorometholone or lotemax (under “Medication (generic name)”) given topically (Route code 5).

A concomitant medication form should be completed for each patient at baseline and each treatment visit. If the concomitant medication form does not specify which eye a particular dose is for then it is assumed that the medication is for the eligible eye/eyes.

Analysis

Censoring within this time to event outcome will occur at failure to respond. This means that a competing risks model is needed, as patients without treatment response are informatively censored (at a lower risk than those who do have treatment response). Therefore a competing risks model will be used with the two competing events being reduction to ≤1 drop and failure to respond. For the second analysis of reduction to 0 drops, the two competing events are reduction to 0 drops and failure to respond.

The competing risks analysis will be performed using the Fine and Gray model. The ‘cmprsk’ package in R will be used for this analysis. The Cumulative incidence plots will be presented. Median time to failing to respond to treatment will be calculated and presented with 95% confidence intervals for each risk. If the algorithm does not converge these cannot be presented.

17.4.4 Optic and Ocular

17.4.4.1 Visual acuity measured by age-appropriate LogMAR assessment

Derivation

This can be found on subsection “Subform Vision assessment and Fundoscopy examination” of the treatment visit CRF under “LogMar score” for the right and left eye.

Analysis

Summary statistics will also be presented in a table for each time point detailing the mean and median values of the LogMar assessment for each treatment arm and overall. This table will report the scores for the eligible eye only. If a patient was eligible based on both eyes then the table will include their worst score and best score in respective columns.

The following analysis will be applied to two analysis sets to account for patients with two study eyes. The first set will include all patients with one study eye, and for the patients with two study eyes, the worst LogMar score of the two eyes will be taken. The second set will include all patients with one study eye, and for the patients with two study eyes, the best LogMar score of the two eyes will be taken.

Mean profile plots and individual plots will be presented. 1-standard error bars will be displayed for each visit on the mean profile plots. This will provide a visual representation of the variation patients may experience in terms of their LogMar score at each scheduled treatment visit. Usually, longitudinal data vs time is plotted (where time goes from the first visit to the final visit). However in joint modelling the relationship between longitudinal data and the event of treatment failure should be examined and so longitudinal data vs time backward is plotted since the event had been occurred. By reversing the time-axis, variation in LogMar of an individual prior to treatment failure will be examined.

The problem of patients having differing amounts of missing data due to treatment failure (and therefore completing follow-up earlier than patients with a later treatment failure time) will be addressed through a more advanced analysis of joint modelling of the longitudinal data of the LogMar score and the time to treatment failure. Joint modelling accounts for the informative missingness in longitudinal LogMar score due to treatment failure (Henderson). If LogMar score is not normally distributed, then a log with base e transformation is used. Patients who did not fail treatment will be censored at the time of the last treatment visit (month 18).

The models will be adjusted for baseline measurements. A random-intercept model for (G)LMM and a shared random effects parameterisation for the association structure is used. Several sensitivity analyses will be carried out:

(1) Missing data: Missing baseline data will be imputed by the mean-value of those available measurements. Intermediate missing data will be imputed by horizontal mean using the patient’s own record; and we adopt the following approach:

(a) If the missing outcome occurred for the first record at time t_{i1}, then it was imputed by the subsequent value (at time t_{i2}); if that value is also missing, then the next actual *observed* value is used.
(b) If the missing outcome occurred at times $t_{i2}, \dots, t_{in_{i-1}}$, it was imputed by the average of available adjacent values.

(2) Distributional assumptions of continuous outcomes: Plot histograms of the baseline values, and quantiles of standardized residuals from a separate LMM fit to assess the normality. Comparisons to log-transformed data (with also log-transformed baseline covariates for consistency) will be made. Where appropriate, the model is re-fitted to the log-transformed data to assess changes in treatment effects. Note that in some cases log-transformations required addition of a positive term if the outcomes took on negative values.

This analysis will be undertaken using the joineR (Philipson, 2012) package in R which uses a maximum likelihood estimation approach (using the EM algorithm) with a Cox PH model for the baseline hazards. Estimates from both the longitudinal and event-time sub-models and the corresponding p-values will be presented.

17.4.4.2 Number of participants with resolution of associated optic nerve or macular oedema (as assessed by slit lamp biomicroscopy or optical coherence tomography)

17.4.4.2.1 Number of participants with resolution of associated optic nerve

Derivation

These can be found on subsection “Subform Intraocular Pressure, Fundoscopy examination and Optical Coherence Tomography” of the treatment visit CRF under the field “Optic nerve (tick if yes)” for both the right and left eyes. If “Normal” is selected then this is defined as not having associated optic nerve, and if “Neovascularisation” or Glaucomatous neuropathy” are selected then this is defined as having associated optic nerve.

If a patient is recorded as having no associated optic nerve but was recorded as having associated optic nerve at the previous visit then this means it has resolved (and classed as a “Yes” for this binary outcome.

The analysis set for this outcome will be patients who have associate optic nerve at baseline or who develop this at any point during the trial.

Analysis

Two analyses will be performed for this outcome: resolution in at least 1 eye, and resolution in both eyes. The numbers and percentages of patients with resolution in at least 1 eye and patients with resolution in both eyes will be presented along with a 95% confidence interval.

In the analysis set for this outcome the percentage of people with resolution of associated optic nerve will be reported (with the denominator being patients who have associate optic nerve at baseline or who develop this at any point during the trial, and the numerator being those with resolution). The number of people who do not experience this at any point during the trial will also be presented.

Alongside this, the number of patients that develop associated optic nerve, is resolved, and then develop this again will be reported.

17.4.4.2.2 Number of participants with resolution of associated macular oedema

Derivation

These can be found on subsection “Subform Intraocular Pressure, Fundoscopy examination and Optical Coherence Tomography” of the treatment visit CRF under the field “Macular oedema present?” for both the right and left eyes.

The analysis set for this outcome will be patients who have macular oedema at baseline or who develop this at any point during the trial.

The data for the ocular co-morbidity defined as ‘Macular Oedema (MO) as gauged clinically and where possible by OCT evidence’ is collected through a fundoscopy assessment and/or OCT assessment. As OCT is more sensitive it can detect macular oedema that is not obvious on fundoscopy. Therefore, if there are conflicting results between fundoscopy and OCT then the OCT result will be used.

Analysis

Two analyses will be performed for this outcome: disease control in at least 1 eye, and disease control in both eyes. The numbers and percentages of patients with disease control

in at least 1 eye and patients with disease control in both eyes will be presented along with 95% confidence intervals.

17.4.4.3 Number of patients who are able to reduce topical or systemic agents for ocular hypertension

Derivation

The question with regards to whether a patient is taking systemic or topical corticosteroids at baseline can be found on the CRF 'Baseline Visit 1 v1.0'. A line listing will also be given for each patient in relation to the total daily dose, route, frequency, start date and end date.

At each visit there is a question as to whether a patient has reduced the topical or systemic agent for ocular hypertension.

Analysis

The number (and percentage) of patients who are able to reduce topical or systemic agents for ocular hypertension will be reported at each time point alongside the appropriate 95% CI alongside a table with a line listing for each patient in relation to the total daily dose, route, frequency, start date and end date.

17.4.4.4 Number of participants with disease control (defined as zero cells, with topical treatment at 12 weeks treatment visit and 24 weeks treatment visit)

Derivation

Disease control is when a patient has a score of "0" for the field "AC cells (SUN)" for 12 or 24 weeks (± 7 days) from registration within each eligible eye and has had at least one topical treatment during this time. AC SUN cells can be found on subsection "Subform Vision assessment and Slit lamp examination" of the treatment visit CRF. Topical treatment is found on CRF "Concomitant Medication Form" and is defined as any treatment given topically (Route code 5). If the concomitant medication form does not specify which eye a particular dose is for then it is assumed that the medication is for the eligible eye/eyes. This will be assessed separately for 3 months (12 weeks ± 7 days i.e. at least 11 weeks) and for 6 months (24 weeks ± 14 days i.e. at least 12 weeks).

Analysis

Two analyses will be performed for this outcome: disease control in at least 1 eye, and disease control in both eyes. The numbers and percentages of patients with disease control in at least 1 eye and patients with disease control in both eyes will be presented with appropriate 95% confidence intervals.

17.4.4.5 Number of participants entering disease remission (defined as zero cells, without topical treatment at 12 and 24 weeks treatment visit)

Derivation

This will be assessed separately for 12 weeks (± 7 days) and 24 weeks (± 7 days). Disease remission is when a patient has a score of “0” for the field “AC cells (SUN)” on the subsection “Subform Vision assessment and Slit lamp examination” of the treatment visit CRF and has not had topical treatment at any point during the 3/6 months. Topical treatment is found on CRF “Concomitant Medication Form” and is defined as any given topically (Route code 5). If the concomitant medication form does not specify which eye a particular dose is for then it is assumed that the medication is for the eligible eye/eyes.

Analysis

Two analyses will be performed for this outcome: disease remission in at least 1 eye, and disease remission in both eyes. The numbers and percentages of patients with disease remission in at least 1 eye and patients with disease remission in both eyes will be presented with appropriate 95% confidence intervals.

In the analysis set for this outcome the percentage of people with disease remission will be reported. The number of people who do not experience this at any point during the trial will also be presented.

Alongside this, the number of patients that enter disease remission more than once (and the number of times disease remission is entered) will be reported.

17.4.4.6 Duration of sustaining inactive disease (zero cells, with or without topical treatment)

Derivation

Inactive disease is when a patient has a score of “0” for the field “AC cells (SUN)” on subsection “Subform Vision assessment and Slit lamp examination” of the treatment visit CRF. This is measured at each follow-up visit and unscheduled visits. The duration of sustaining inactive disease is calculated by summing the total consecutive months in which a patient has a score of 0 AC SUN cells. If a patient has a score of 0 AC SUN cells for just one visit then this is not classed as “sustained inactive disease”, i.e. a patient must have *consecutive* visits with a score of 0.

Analysis

A random intercept model will be used to account for the correlation between eyes within patients. The outcome variable will be duration of sustaining inactive disease and the covariate will be eye.

Treatment effect estimates and confidence intervals will be estimated in SAS using the procedure “PROC MIXED”. The options ‘solution’ and ‘cl’ will be added to give the parameter estimates and confidence intervals, respectively.

17.4.4.7 Failure to reduce eye drops to 2 drops/day by or at the 12 weeks visit

Derivation

This only applies to a sub-set of patients who are on greater than 2 drops at registration, recorded in the field “AC cells (SUN)” on the subsection “Slit lamp examination” and have a recording of 2 or less drops at any visit following this.

Analysis

The numbers and percentages of patients with reduction to 2 drops will be presented with appropriate 95% confidence intervals.

17.4.5 Quality of Life assessment (CHQ and CHAQ)

17.4.5.1 Childhood Health Questionnaire (CHQ)

Derivation

Refer to the PDF held on the CD "Child Heath Questionnaire (CHQ) Scoring and Interpretation Manual" (pages 32 – 53 regarding the CHQ-PF50) (Child Heath Questionnaire (CHQ) Scoring and Interpretation Manual) for details on how to derive the overall score for the CHQ, as this cannot be reproduced within this SAP due to copyright.

Analysis

This will be analysed using joint modelling as described in section 0.

Mean profile plots and individual plots will be presented. 1-standard error bars will be displayed for each visit on the mean profile plots. This will provide a visual representation of the variation patients may experience in terms of their overall CHQ score at each scheduled treatment visit.

17.4.5.2 Childhood Health Assessment Questionnaire (CHAQ)

Derivation

This is the overall score on the CRF "CHAQ UK Questionnaire" (CHAQ). Any 'component' questions are scored as follows:

- Without any difficulty = 0
- With some difficulty = 1
- With much difficulty = 2
- Unable to do = 3

If not answered or "Not applicable" is selected then score is left blank for this question.

This questionnaire is made up of 10 categories:

1. Dressing & personal care
2. Getting up
3. Eating

4. Walking
5. Hygiene
6. Reach
7. Grip
8. Activities
9. Pain
10. General evaluation

Each category can be given a score out of 3. These are made up from the component questions and whether anything from the “Aids or devices” and/or “Help from another person due to illness” for the relevant category is selected.

Devices associated with each category:

- Dressing & personal care
  - Devices used for dressing (button hook, zipper pull, long handled show horn etc.)
- Getting up
  - Special or built up chair
- Eating
  - Built up pencil or special utensils
- Walking
  - Walking stick
  - Walking frame
  - Crutches
  - Wheelchair
- Hygiene
  - Raised toilet seat
  - Bath seat
  - Bath rail
  - Long-handled appliances in bathroom
- Reach

   - Long-handled appliances for reach
- Grip
   - Jar opener
- Activities

If anything is selected under these sections then the score is 2. The overall score for each category is determined by the highest score given within that category.

For example, if someone selected “with some difficulty” for all the questions in the “dressing and personal care” category, and also selected “Devices used for dressing (button hook, zipper pull, long handled show horn etc.)” then the score for the “dressing and personal care” category would be 2. If someone selected “unable to do” for anything within the “getting up” section then the score for this category would be 3.

The “Pain” and “General evaluation” categories are scored as a number between 0 and 100. To convert this into a scale of 0 to 3, divide the score by 100 and multiply by 3 and round to the nearest 1 decimal place.

The overall index is calculated by summing the overall scores for each of the categories and dividing by the number of categories answered. This will give a score between 0 and 3.

Analysis

This will be analysed using joint modelling as described in section 0.

Mean profile plots and individual plots will be presented. 1-standard error bars will be displayed for each visit on the mean profile plots. This will provide a visual representation of the variation patients may experience in terms of their CHAQ score at each scheduled treatment visit.

17.4.6 American College of Rheumatology (ACR) Pedi core set criteria: at ACR30, ACR50, ACR70, ACR90 and ACR100 levels

Derivation

The 6 paediatric core set criteria assessed at each study visit are:

- Physician global assessment of disease activity (10 cm visual analogue scale).
- Parent/patient assessment of overall well-being (10 cm visual analogue scale).
- Functional ability (Childhood Health Assessment Questionnaire, CHAQ).
- Number of joints with active arthritis.
- Number of joints with limited range of movement.
- Erythrocyte sedimentation rate.

The ACR Paediatric 30, 50, 70, 90 and 100 levels (Giannini, 1997) are defined as 30%, 50%, 70%, 90% and 100% improvement respectively in a minimum of three variables in the core set with worsening of one variable by no more than 30% as defined in the ACR criteria.

Physician global assessment of disease activity (10 cm visual analogue scale)

This can be found on subsection “Subform Urinalysis Test, Physical Exam, Vital Signs and Physician global assessment of disease” of the treatment visit CRF under the field “Physician global assessment of disease activity”.

Parent/patient assessment of overall well-being (10 cm visual analogue scale)

This can be found on the CRF “CHAQ UK Questionnaire” under the last question “General evaluation”.

Functional ability (Childhood Health Assessment Questionnaire, CHAQ)

See analysis section of 17.4.5.2 for details on how to derive the overall score for the CHAQ.

Number of joints with active arthritis

This can be found on subsection "Subform Recorded pattern of joint involvement on examination and Spinal assessments" of the treatment visit CRF. Tabulate number of joints selected from the "Active" column.

Number of joints with limited range of movement

This can be found on subsection "Subform Recorded pattern of joint involvement on examination and Spinal assessments" of the treatment visit CRF. Tabulate number of joints selected from the "Limited" column.

Erythrocyte sedimentation rate

This can be found on subsection "Subform Haematological and Biochemical Assessments" of the treatment visit CRF.

Analysis

Patients may have differing amounts of missing data due to treatment failure (and therefore completing follow-up earlier than patients with a later treatment failure time). This issue will again be addressed through joint modelling of the longitudinal data of the ACR30, ACR50, ACR70, ACR90 and ACR100, and the time to treatment failure.

Patients who did not fail treatment will be censored at the time the last treatment visit (month 18). As the ACR levels are binary outcomes, this analysis will be undertaken using the JMbayes (Rizopoulos) package in R from which the log odds ratio with 95% confidence interval from the longitudinal model will be estimated. This uses a Bayesian MCMC estimation approach, with a spline approximated baseline hazard function.

We do not include baseline adjustment in the model as ACR records improvement from baseline. If a joint model could not be fitted as specific requirements of the JMbayes package are not met (i.e. a fitted separate model for the longitudinal outcome estimated using penalized quasi-likelihood methods using the `glmmPQL()` function), then we fit an

alternative model using a more contemporary R package (`glmer()` from the `lme4` package) to estimate the treatment effect on longitudinal ACR.

The number and percentage of patients with each ACR response will be presented for each time point as a bar graph. Mean profile plots will be presented with 1-standard error bars displayed for each visit. Summary statistics will also be presented in a table for each time point detailing the number and percentage of patients with ACR responses.

17.4.7 Number of participants requiring change in biologic /disease-modifying anti-rheumatic drugs (DMARDs) therapy due to disease flare of their arthritis or failure to respond to treatment of arthritis

Derivation

This can be found on subsection “Subform Trial Intervention” of the treatment visit CRF under the field “Has participant required a change in biological/DMARD therapy since the last visit due to a failure to respond for arthritis?” and is given as a binary “Yes/No” response. If a patient has at least one change at any time during the trial then they are classed as a “yes”. Only patients who don’t have a change at any point are classed as a “no”.

Analysis

The data will be summarised by the number (and percentage) of patients that required change in biologic/Disease-modifying anti-rheumatic drugs (DMARDs) therapy due to failure to respond from arthritis. Individual Information on change in biologic or disease-modifying anti-rheumatic drug will be listed by patient as line listings.

17.4.8 Number of participants undergoing flare of arthritis

Derivation

For this secondary outcome, ‘disease flare’ refers to a flare of arthritis rather than the eye. The definition of ‘disease flare’ is a worsening of 30% or more in 3 or more of the 6 variables of the JIA core set , with no more than one variable improving by 30% or more. In addition, the following minimum worsening contingencies apply: if either the number of active joints or the number of joints with limited range of motion are included in the calculation of "flare" then there must be a worsening of at least two joints. If the Physician’s or parent/legal guardian global rating scores are used in the definition of "flare" then there

must be a worsening of at least 2 units on the 10 unit scales. If the ESR is used in the definition of "flare" then the second value for the ESR used in the calculation must be above the upper limit of normal for the ESR. For subjects with sJIA, presence of spiking fever (>38°C, occurring on at least 2 consecutive days) due to sJIA will be considered alone as flare (Brunner, 2002). If a patient has at least one disease flare at any time during the trial then they are classed as a "yes". Only patients who don't experience a disease flare at any point are classed as a "no".

Analysis

The data will be summarised by the number (and percentage) of patients with at least one disease flare.

17.4.9 Number of participants in remission on and off medication of their JIA

17.4.9.1 Number of participants in remission on medication of their JIA

Derivation

Criteria for inactive disease of their JIA is defined as per Wallace 2004:

- No joints with active arthritis (See "Subform Recorded pattern of joint involvement on examination and Spinal assessments" of the treatment visit CRF)
- No fever, rash, serositis, splenomegaly, or generalized lymphadenopathy attributable to JIA – this component only applies to systemic JIA patients and, given that these patients are not enrolled into APTITUDE, we do not need to consider this for the analysis.
- No active uveitis (AC cells score of 0 – See "Subform Binocular indirect opthalmoscope and Slit lamp exam" of the treatment visit CRF under "AC Cells (SUN)" for the right and left eye)
- Normal ESR or CRP (if both are tested, both must be normal) (See "Subform Haematological and Biochemical Assessments" of the treatment visit CRF. It can also be found here whether the assessment result was "Normal", "Abnormal" or "Not done", and also whether the result was "Clinically significant" (only applicable if the result was abnormal).). Only those indicated as 'normal' will count here.

- Physician's global assessment of disease activity indicates no disease activity (See derivation section of 17.4.6 for details on how to derive this)

Clinical remission of JIA whilst on medication is defined as per Wallace 2004:

- The criteria for inactive disease must be met for a minimum of 6 continuous months while the patient is on (anti-rheumatic, anti-inflammatory* and anti-uveitis) medication in order for the patient to be considered to be in a state of clinical remission on medication.

* Anti-inflammatory treatments taken to treat the arthritis rather than those used for therapeutic symptom relief (e.g. ibuprofen, etc) or for other reasons such as a headache.

Only patients that were <u>on</u> medication for at least 6 months will be included in this analysis as this is the minimum amount of time required to assess for clinical remission <u>on</u> medication of JIA.

As per trial protocol, doses of trial treatment (tocilizumab) are to be taken every 14 days ($\pm$3 days) so therefore the lower limit for the 6-month time period is 11 days * 2 (per month) * 6 months = 132 days.

During the treatment phase of the trial all patients will be classed as <u>on</u> medication as they will be taking Tocilizumab. For conmeds taken during the 1-month follow-up phase all conmeds (recorded on conmeds CRF) that (1) have a start date on or prior to the last treatment visit date (at 6 months) and have an end date after the last treatment visit date or are ongoing; or (2) have a start date after the last treatment visit date will be categorised as:

(i) anti-rheumatic (classified as a medication for treatment of JIA);
(ii) anti-inflammatory for treatment of arthritis (classified as a medication for treatment of JIA);
(iii) anti-inflammatory for therapeutic reasons or non-treatment of arthritis (<u>not</u> classified as a medication for treatment of JIA);

(iv) Anti-uveitis (classified as a medication for treatment of JIA)

(v) None of the above (not classified as a medication for treatment of JIA).

Line listings of all conmeds taken during follow-up will be reviewed by the Chief Investigators to confirm classifications as above. These will be used to determine how many days after the last treatment visit date (at 6 months) the patient is classed as still 'on medication'.

Of those patients that have been on medication for at least 6 months, they will be assessed for inactive disease of their JIA. A 'Yes/No' variable will be created for each of the inactive disease components at all visits whilst on medication. A 'Yes' to all components at a visit indicates inactive disease of their JIA at that visit. Inactive disease at all visits within a 6-month period whilst on medication.

ESR or CRP values just outside the normal range which are not deemed to be clinically significant are nothing to be concerned with in terms of the care for the patient. Therefore, we will undertake a sensitivity analysis to classify 'abnormal but not clinically significant' ESR and CRP values as 'normal' in the definition of inactive disease of their JIA.

Analysis

For both the main analysis and sensitivity analysis, the number and percentages of patients that achieve clinical remission of JIA whilst on medication will be presented with appropriate 95% confidence intervals. The number of patients that were not on medication for at least 6 months will also be presented.

17.4.9.2 Number of participants in remission off medication of their JIA

Clinical remission of JIA whilst off medication is defined as per Wallace 2004:

- The criteria for inactive disease (see definition in section 17.4.9.2 above) must be met for a minimum of 12 continuous months while off all anti-rheumatic, anti-inflammatory* and anti-uveitis medications in order for the patient to be considered to be in a state of clinical remission off medication.

* Anti-inflammatory treatments taken to treat the arthritis rather than those used for therapeutic symptom relief (e.g. ibuprofen, etc) or for other reasons such as a headache.

Patients will not be able to assessed for inactive disease for a minimum of 12 months with the treatment phase being 6 months and the follow-up phase being 1 month so clinical remission of JIA whilst off medication is unable to be analysed.

17.4.10 Juvenile Arthritis Disease Activity Score (JADAS)

Derivation

The Juvenile Arthritis Disease Activity Score (JADAS) is comprised of four components[13]:

- physician global assessment of disease activity,
- parent/patient global assessment of well-being,
- active joint count, in 27, 71 or 10 joints,
- erythrocyte sedimentation rate (ESR).

The JADAS is calculated as a sum of scores from its four components detailed below, giving global scores of 0-57, 0-101 and 0-40 for the JADAS-27, JADAS-71 and JADAS-10 respectively.

Physician global assessment of disease activity

See derivation section of 17.4.6 for details on how to derive this.

Parent/patient global assessment of well-being

See derivation section of 17.4.6 for details on how to derive this.

Active joint count, in 27, 71 or 10 joints

This can be found on subsection "Subform Recorded pattern of joint involvement on examination and Spinal assessments" of the treatment visit CRF under the "Active" column.

27 Joints

The joints considered are:

1. Cervical spine
2. Left elbow
3. Right elbow
4. Left wrist
5. Right wrist
6. Left MCP 1
7. Right MCP 1
8. Left MCP 2
9. Right MCP 2
10. Left MCP 3
11. Right MCP 3
12. Left PIP 1
13. Right PIP 1
14. Left PIP 2
15. Right PIP 2
16. Left PIP 3
17. Right PIP 3
18. Left PIP 4
19. Right PIP 4
20. Left PIP 5
21. Right PIP 5
22. Left hip
23. Right hip
24. Left knee
25. Right knee
26. Left ankle
27. Right ankle

The total number of joints is summed for the relevant joints to obtain a value between 0 and 27. This corresponds to the JADAS-27 score.

<u>71 joints</u>

All joints are considered within this, excluding ‘Thoracic Spine’, ‘Lumbar Spine’ and ‘Sacroiliac Joints’. The total number of joints is summed to obtain a value between 0 and 71. This corresponds to the JADAS-71 score.

10 joints

All joints are considered within this, excluding ‘Thoracic Spine’, ‘Lumbar Spine’ and ‘Sacroiliac Joints’. The total number of joints are summed however if a patient has >10 active joints then the total number of joints is 10. This will give a value between 0 and 10. This corresponds to the JADAS-10 score.

Erythrocyte sedimentation rate (ESR)

See derivation section of 17.4.6 for details on how to derive this. For this outcome, ESR values < 20mm/hour should be converted to 20* and ESR values >120mm/hour should be converted to 120. The ESR values should then be normalised to a 0-10 scale by:

$$\frac{ESR\ (mm/hour)\ -20}{10}$$

*This formula is taken from Consolaro A, Ruperto N, Bazso A et al. (2009), however it is assumed that ESR values < 20mm/hour should be converted to 20, rather than 0 as stated in Consolaro A, Ruperto N, Bazso A et al, 2009.

Analysis

This will be analysed using joint modelling as described in the analysis section of 17.4.4.1.

Mean profile plots will be presented with 1-standard error bars displayed for each visit. This will provide a visual representation of the variation patients may experience in terms of their overall JADAS-27, JADAS-71 and JADAS-10 score at each scheduled treatment visit.

17.4.11 Number of participants with minimum disease activity

Derivation

Minimum disease activity is defined for those with Oligoarthritis and Polyarthritis. This information can be found on CRF “Inclusion and Exclusion under the field “ILAR

classification”. As this is only defined for Oligoarthritis and Polyarthritis, patients with Systemic arthritis, Psoriatic arthritis, Enthesitis-related arthritis or Undifferentiated arthritis are excluded.

Table 1 (Magni-Manzoni, 2008) shows the threshold values that defines minimum disease activity. If a patient with oligoarthritis/polyarthritis has values less than or equal to the values in the corresponding column at *any* treatment visit then they are defined as “Yes” for minimum disease activity. If they have values greater than any of the threshold values at *all* treatment visits then they are defined as “No” for minimum disease activity.

TABLE 1: Threshold values for juvenile idiopathic arthritis activity measures that best discriminated between states of high and minimal disease activity obtained through the area under the receiver operating characteristic curve analysis.

	Oligoarthritis (n = 248)	Polyarthritis (n = 281)
Physician's global assessment of disease activity*	≤2.5	≤3.4
Parent's global assessment of well-being*	≤0.3	≤2.1
Parent's pain assessment*	≤0.9	≤2
Childhood Health Assessment Questionnaire score†	≤0.25	≤0.25
Number of swollen joints	0	≤1
Number of tender joints	0	≤1
Number of joints with restricted motion	0	0
Number of active joints	0	≤3
Morning stiffness, minutes	0	0
Erythrocyte sedimentation rate, mm/hour‡	≤16	≤20
C-reactive protein level, mg/dl§	≤0.41	≤0.9
White blood cells, $\times 10^9$/liter	≤8.2	≤7.1
Hemoglobin, gm/liter	>121	>119
Platelets, $\times 10^9$/liter	≤392	≤379

* Range 0 (best) to 10 (worst).
† Range 0 (best) to 3 (worst).
‡ Normal value <15 mm/hour.
§ Normal value <0.4 mg/dl.

Each of the measures can be found as follows:

- Physician global assessment: subsection "Subform Urinalysis Test, Physical Exam, Vital Signs and Physician global assessment of disease" of the treatment visit CRF under the field "Physician global assessment of disease activity".
- Parent global assessment: CRF "CHAQ UK Questionnaire" under the last question "General evaluation". This is entered as a value of 0 to 100 on the database so divide by 10 for a range of 0 to 10.
- Parent's pain assessment: CRF "CHAQ UK Questionnaire" under the question "Pain". This is entered as a value of 0 to 100 on the database so divide by 10 for a range of 0 to 10.
- CHAQ score: See analysis section of 17.4.5.2 for details on how to derive the overall score for the CHAQ.
- Number of swollen joints: subsection "Subform Recorded pattern of joint involvement on examination and Spinal assessments" of the treatment visit CRF. Tabulate number of joints selected from the "Swollen" column.
- Number of tender joints*
- Number of joints with restricted motion: subsection "Subform Recorded pattern of joint involvement on examination and Spinal assessments" of the treatment visit CRF. Tabulate number of joints selected from the "Limited" column.
- Number of active joints: subsection "Subform Recorded pattern of joint involvement on examination and Spinal assessments" of the treatment visit CRF. Tabulate number of joints selected from the "Active" column.
- Morning stiffness
- Erythrocyte sedimentation rate: subsection "Subform Haematological and Biochemical Assessments" of the treatment visit CRF under "Biochemical Assessments".
- C-reactive protein level: subsection "Subform Haematological and Biochemical Assessments" of the treatment visit CRF under "Haematological Assessments".
- White blood cells: subsection "Subform Haematological and Biochemical Assessments" of the treatment visit CRF under "Haematological Assessments".

- Haemoglobin: subsection “Subform Haematological and Biochemical Assessments” of the treatment visit CRF under “Haematological Assessments”.
- Platelets: subsection “Subform Haematological and Biochemical Assessments” of the treatment visit CRF under “Haematological Assessments”.

Analysis

Mean profile plots will be presented for each measure of minimum disease activity for Oligoarthritis and Polyarthritis. 1-standard error bars will be displayed for each visit on the mean profile plots. For each time point the mean, SD, median, IQR and range will be presented for each measure of minimum disease activity.

The data will be summarised by the number (and percentage) of patients with at least one case of minimum disease activity.

Proportions of patients with at least one case of minimum disease activity will be presented for each time point. The number of patients with at least one case of minimum disease activity (and the amount of cases of minimum disease activity) will also be reported.

18 Missing data and withdrawals

The proportion of missing data will be presented overall for each outcome with further detail as to the reason as appropriate.

Participants that withdraw from the trial completely (providing they do not withdraw consent to use their data), will contribute outcome data until the point at which they withdrew using an ITT approach.

For each participant that discontinues treatment before the 12 week assessment of primary outcome, a clinical decision will be made as whether the patient should be classified as a ‘treatment response or not’. This decision will be made by both the Chief Investigators (Professor Beresford and Professor Ramanan) and also Professor Dick. Professor Ramanan will no take part in decisions made about patients from Bristol.

19 Additional analyses

The following sensitivity analyses will be conducted:

1) All participants that have withdrawn from treatment will be treated as a failure to respond at the time of withdrawal.

2) Loss to follow-up: In the primary analysis of primary outcome we have classed the patients that are lost to follow-up as withdrawals assuming that they are non-informative. The reasons for loss to follow-up, where available, will be reviewed by Prof Michael Beresford (Co-Chief Investigator) and Prof Andrew Dick (Ophthalmology expert on TMG) to see to see whether they think any might be related to prognosis. If any are deemed to be related, a sensitivity analysis will be undertaken assuming these patients to be a non-treatment response at the time of last recorded visit.

3) Incorrectly identified to be a treatment response: If there are any patients that were wrongly identified to be treatment response by the assessing physician they will be classed as a withdrawal at this time.

4) ‘Number of participants in remission on and off medication of their arthritis’ will be analysed as per ‘Number of participants in remission on and off medication of their JIA (Wallace 2004)’ with the criteria in the Wallace 2004 paper that apply to uveitis omitted to only use those that apply to arthritis. The criteria to be omitted are as follows (i) active uveitis and (ii) anti-uveitis medications. The outcome will be analysed as per section 17.4.9.

20 Safety Evaluations

All results summaries will be presented to 1 decimal place.

20.1 Data sets analysed

Patients that withdrew consent for trial continuation will contribute outcome data up until the point of withdrawal unless the patients' parents/guardians specifically request that the data are not to be used.

The safety analysis data set will contain all participants that are registered and received at least one dose of trial medication. As per the APTITUDE trial protocol (all versions), adverse event data should only be collected up to 30 days following cessation of treatment. Any AEs recorded with an onset date after this 30-day cut off will be excluded. Any AEs with a missing onset date will be included.

For safety outcomes, any dates that have the month and year recorded but not the specific day will be imputed as being the middle of the month (the 15th). If an imputed date proceeds the registration date then take the registration date. If an imputed date exceeds the last treatment visit date + 30 days then take the last treatment visit date + 30 days.

Patients to be excluded from the safety analysis population will be defined in template 'ST001TEM04 Protocol deviations and population exclusions template' and this will be agreed and approved prior to any release of final registration codes.

20.2 Presentation of the data

AEs are captured on the CRFs as free-text. The APTITUDE Macro 4 database automatically populates the MedDRA codes based on the AE free-text description field. If there is not a direct match the MedDRA codes will not be populated. The unmatched AEs will be categorised by the Data Manager using MedDRA coding with Chief Investigator input and subsequently signed off by Chief Investigator once complete, prior to unblinding the patients included in the analysis.

All adverse events (AEs) and serious adverse events (SAEs) will be presented separately and overall at the MedDRA SOC level. No formal statistical testing will be undertaken. The safety population will be used for these summaries listed below.

Adverse event data will be presented at the MedDRA SOC level in as follows: (i) Serious adverse events (SAEs) and (ii) All adverse events (AEs) inclusive of any that are also SAEs.

Additionally, results for (iii) Non-serious adverse events will be derived but will only be input directly into the EUDRACT database and not into the final statistical analysis report. Results for (i) will also be input into the EUDRACT database.

The number of events and number (and percentage) of patients experiencing each AE (split by MedDRA SOC and PT) and a cross-tabulation of AE severity (mild, moderate, severe, missing) by relationship to study drug (Unrelated (unrelated, unlikely); Related (possibly, probably, almost certainly); Missing) will be presented. Registration numbers with frequency of occurrence will be listed next to each AE MedDRA PT in the appropriate cells within this table.

The incidence rates of total numbers of AEs/SAEs will be calculated in person-years. This is defined as the total number of AEs/SAEs divided by the total exposure time in years (calculated as total days / 365.25) for all participants. A table will be presented showing:

- Total years
- Total AEs
- AE rate per patient year
- 95% confidence interval* for AE rate per patient year
- Total SAEs
- SAE rate per patient year
- 95% confidence* interval for SAE rate per patient year.

* If the estimate is based on very few sample cases, the confidence interval can include a negative lower confidence limit. For these cases, the lower limits should be set to 0.

Each SAE will be presented in the form of line listings detailing:

- SAE number
- SAE report date
- Age
- Gender
- Weight at baseline
- Weight at SAE assessment date
- Height
- Location of CRF
- AE description (verbatim and MedDRA SOC/PT/LLT)
- SAE Duration (days)
- Severity
- Seriousness
- Action taken
- Outcome
- Expectedness to **Tocilizumab** (PI and Chief Investigator assessments)
- Relationship to **Tocilizumab** (PI and Chief Investigator assessments)
- Cause
- SAE onset date
- Onset timing since last dose of IMP
- Study treatment at time of even
- Drug concentration at baseline
- Dose at last administration
- Duration of IMP
- Patient status
- Whether unblinded
- Last dose of IMP
- Whether withdrew from drug as a result of AE.

SAEs which are SUSARs will be identified as such.

Each SAE has an ‘initial report’ done. If the SAE has not yet been resolved the ‘resolved date’ is left blank. Later, a ‘follow-up report’ or a ‘final report’ captures the ‘resolved’ date and final outcome. Therefore, the latest report will be taken and presented as the line listings.

The total duration of treatment (days) will be calculated per patient and will be summarised by mean, SD, median and range (maximum and minimum values) regardless of whether the data are skewed or not for each treatment group.

The duration of treatment (days) will also be categorised and summarised by frequencies and percentages. The categories will be displayed as separate batches of 28-day intervals: ‘1-28’, ’29-56’, ’57-112’ etc.

To ensure quality control, an independent statistician will follow this SAP to independently program the safety analysis from the raw data. Any discrepancies found will be discussed with the trial statistician to resolve. No programming will be shared or shown between the statisticians. The independent statistician will also check the report against their output.

21 References

1. *International Conference on Harmonisation. Topic E3 Structure and content of clinical study reports.(CPMP/ICH/137/95). 1995*
2. *International Conference on Harmonisation. Topic E9 Statistical Principles for Clinical Trials (CPMP/ICH/363/96). 1998.*
3. *Guidance on Statistical Analysis Plans.*
4. *Schulz KF, Altman DG, Moher D, for the CONSORT Group. CONSORT 2010 Statement: updated guidelines for reporting parallel group randomised trials. BMJ 2010;340:c332..*
5. *Shuster JJ: Diagnostics for assumptions in moderate to large simple clinical trials: do they really help? Stat Med 2005, 24(16):2431–2438.*
6. *Campbell H, Dean CB. The consequences of proportional hazards based model selection. Statistics in Medicine. 2013*
7. *Raab GM, Day S, Sales J: How to select covariates to include in the analysis of a clinical trial. Control Clin Trials 2000, 21(4):330–342.*
8. *Simon R. Optimal two-stage design for phase II clinical trials. Controlled Clinical Trials 1989; 10:1--10. DOI: 10.1016/0197-2456(89)90015-9.*
9. *Jovic G, Whitehead J. An exact method for analysis following a two-stage phase II cancer clinical trial. Statistics in medicine 2010; 29(30):3118-25.*
10. *Clopper CJ, Pearson ES. The use of confidence or fiducial limits illustrated in the case of the binomial. Biometrika 1934; 26:404-413.*
11. *Freeman PR: The performance of the two-stage analysis of two treatment, two-period crossover trials. Stat Med 1989, 8(12):1421–1432.)*
12. *Kahan BC: Bias in randomised factorial trials. Stat Med 2013, 32(26):4540–4549.*
13. *Henderson R, Diggle PJ, Dobson A. Joint modelling of longitudinal measurements and event time data. Biostatistics; 1: 465–480*
14. *Philipson P, Sousa I, Diggle PJ, Williamson PR, Kolamunnage-Dona R, Henderson R. Package " joineR " 2012.*
15. *Child Heath Questionnaire (CHQ) Scoring and Interpretation Manual*
16. *Childhood Health Assessment Questionnaire: https://www.niehs.nih.gov/research/resources/assets/docs/chaq_instructions_508.pdf*
17. *Giannini EH, Ruperto N, Ravelli A, Lovell DJ, Felson DT, Martini A. Preliminary definition of improvement in juvenile arthritis. Arthritis Rheum 1997;40:1202-1209.*
18. *Brunner HI, Lovell DJ, Finck BK, Giannini EH. Preliminary definition of disease flare in juvenile rheumatoid arthritis. J Rheumatol 2002; 29(5):1058-1064.*
19. *Consolaro A, Ruperto N, Bazso A et al. Development and validation of a composite disease activity score for juvenile idiopathic arthritis. Arthritis Rheum 2009; 61(5):658-666.*
20. *Magni-Manzoni S, Ruperto N, Pistorio A, Sala E, Solari N, Palmisani E, Cugno C, Bozzola E, Martini A, Ravelli A. Development and validation of a preliminary definition of minimal disease activity in patients with juvenile idiopathic arthritis. Arthritis Rheum 2008;59:1120-1127.*
